# Phosphodiesterase 4 inhibitor activates AMPK-SIRT6 pathway to prevent aging-related adipose deposition induced by metabolic disorder

**DOI:** 10.18632/aging.101559

**Published:** 2018-09-18

**Authors:** Zhuoran Wang, Yaru Liang, Lu Zhang, Nannan Zhang, Qingfei Liu, Zhao Wang

**Affiliations:** 1 MOE Key Laboratory of Protein Sciences, School of Pharmaceutical Sciences, Tsinghua University, Beijing 100084, P.R. China; * Equal contribution

**Keywords:** rolipram, aging, adipose deposition, AMPK, SIRT6

## Abstract

Rolipram is a selective phosphodiesterase 4 (PDE4) inhibitor that exerts a variety of effects, including anti-inflammatory, immunosuppressive, and anti-tumor effects. The aim of this study was to investigate the effect of rolipram on metabolic disorder and its underlying mechanisms. Metabolic disorder was induced in 8-week-old wild type BABL/c mice by administration of D-galactose for 4 weeks. Simultaneously the mice were administered vehicle or rolipram. Alternatively, beginning at 3 or 21 months, the mice were administered db-cAMP for 3 months, with or without a high-fat-diet (HFD) to induce metabolic disorder. In both models, better metabolic function was observed in rolipram-treated mice. Rolipram reduced adipose deposition and inflammation and reserved metabolic disorder. Treatment with rolipram increased the AMPK phosphorylation and SIRT6 levels in the liver and kidney while reducing NF-κB acetylation. In vitro, these effects were blocked by suppression of SIRT6 expression using specific siRNA. Increased cAMP levels reduced excessive adipose deposition, and improved adipose distribution in presenile mice. These findings provide a promising strategy for the treatment of aging-related metabolic dysfunctions and suggest that selective PDE4 inhibitors may be useful agents for the treatment of aging-related metabolic diseases.

## Introduction

Chronic aging-related diseases, such as diabetes and cardiovascular diseases are strongly associated with metabolic disorders and ultimately shorten health and life span [1]. Dysregulation of metabolic functions can be induced by multiple factors, including genetic and environmental influences. For example, abnormal glucose metabolism or a high-fat diet (HFD) can negatively affect the aging process and lead to reduced survival [2].

Rolipram is a well-studied antidepressant drug that failed in clinical trials due to its significant side effects [3,4]. Notably, rolipram was also found to have a potentially protective effect against Alzheimer's disease [5–8] and may also be useful for the treatment of ischemia injury [9–12] and for rescue after spinal cord injury [13,14]. Acting as a specific phosphodiesterase 4 (PDE4) inhibitor, rolipram increases cellular levels of the second messenger cAMP [15,16], the effect of which varies depending on the effectors [17–20]. Rolipram was shown exert partial protection effect against diet-induced obesity and glucose intolerance [21,22]. It is therefore possible that cAMP activators or PDE4 inhibitors may be useful for treating metabolic diseases and other aging-related diseases in humans.

AMP-activated phosphate kinase (AMPK) is an energy sensor that plays an essential role in metabolic regulation [23]. Activation of AMPK is via energy depletion reflected by an increased AMP/ATP ratio or via increased intracellular Ca^2+^ leading to activation of CaMKKβ [24,25]. AMPK is also highly associated with the sirtuin family of nicotinamide adenine dinucleotide (NAD)-dependent protein deacetylases, which have been implicated in nutritional metabolism and life span regulation [22,26–28]. Within mammalian genomes, there are seven sirtuin genes (*SIRT1*-*SIRT7*), among which inactivation of the *SIRT6* gene in mice leads to premature aging-like phenotypes and dramatically shortened lifespan. These phenotypes include bone mineral density defects, spinal curvature abnormalities, loss of subcutaneous fat, lymphocyte attrition, and severe metabolic defects [29]. In addition, male mice overexpressing SIRT6 have a longer lifespan than wildtype mice [30]. SIRT6 attenuates signaling by the pro-inflammatory transcription factor nuclear factor-kappaB (NF-κB) through H3K9 chromatin deacetylation, and hyperactive NF-κB signaling may contribute to premature and normal aging [31]. Notably, there is reportedly a correlation between the function of NF-κB and those of cAMP [18,32,33]. This suggests cAMP activators or PDE4 inhibitors could potentially exert beneficial effects through suppression inflammation [3].

Metabolic disorders often lead to chronic inflammation, which is very harmful for our heath span [34,35]. Although aging-associated metabolic dysregulation and chronic inflammation typically do not yield a clear diagnosis of a specific disease, they are key features of a variety of aging-related diseases [36]. The aim for our study was to investigate the effect of rolipram on metabolic disorder and chronic inflammation, during the aging process. Rolipram was previously shown to potently reduce weight gain in a diet-induced obesity mice model [22] and to prevent Alzheimer's disease [7], but the potential of a specific PDE4 inhibitor to serve as an anti-aging drug by improving metabolic functions has not yet been investigated.

## RESULTS

### Rolipram treatment improves adipose distribution in presenile mice

**Figure 1:**
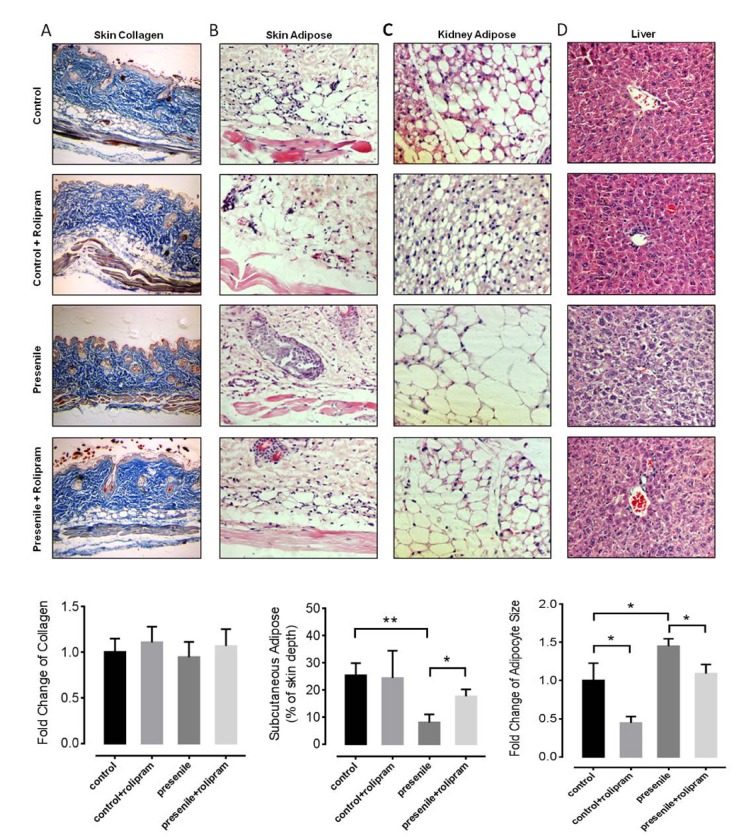
**Rolipram improves adipose distribution in presenile mice.** (**A**) Masson’s trichrome staining of the skin of mice with or without rolipram treatment. The blue staining shows the collagen content (original magnification: 10x in both images). (**B**-**D**) Hematoxylin and eosin staining of the skin (**B**), kidney adipose (**C**) and liver tissue (**D**) in mice with or without rolipram treatment. The unstained vacuoles are the adipose tissue (original magnification: 40x in both images).

To test whether rolipram administration would positively influence on the aging-associated phenotypes, we prepared an experimental presenile model using male mice in which a metabolic disorder was induced (galactosemia induced by 30 days of D-galactose administration). We treated the mice with or without rolipram (2 mg/kg body weight per day intraperitoneally). To investigate the role of rolipram in the pathology seen in presenile mice, histochemical staining was utilized to observe aging-associated phenotypes in multiple mice tissues. One aging-associated phenotype, reduced skin elasticity is caused by loss collagen and subcutaneous adipose. In our model, presenile mice lost the subcutaneous adipose tissue, though the collagen content did not significantly change (Figure 1A). Mice treated with rolipram retained their subcutaneous adipose (Figure 1B), contributing to better skin elasticity. To our surprise, adipocytes around the kidneys of rolipram treated mice were significantly smaller than untreated presenile mice (Figure 1C). Rolipram also decreased adipose deposition in the livers of presenile mice (Figure 1D). These data suggest that rolipram reduces excessive adipose deposition in organs, such as the liver and kidney while maintaining beneficial adipose tissue. In this way, rolipram improves the adipose distribution in presenile mice.

### Rolipram reverses metabolic disorder

**Figure 2:**
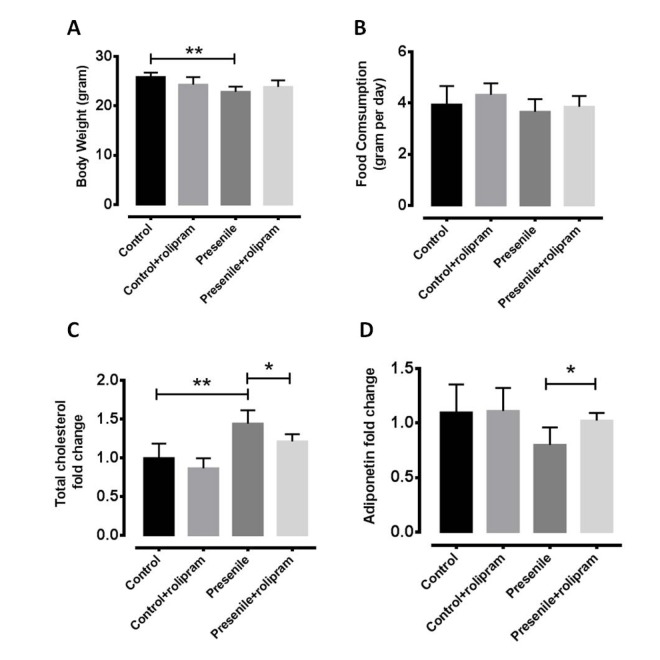
**Rolipram reverses metabolic disorders.** (**A**) Body weight and (**B**) food intake in normal and presenile mice, with or without rolipram treatment. (**C**) Serum total cholesterol level and (**D**) hepatic adiponectin levels in rolipram-treated and untreated presenile mice.

To test whether rolipram can delay the functional declines of presenile mice, we analyzed behavioral performances in open-field and rota-rod experiments. The locomotors activities were nearly completely restored in presenlie mice administered dibutyryl (db)-cAMP (Figure S1A). Further, the db-cAMP-treated mice exhibited significantly improved motor coordination as well as learning and memory (Figure S1B to 1D). There were no significant changes in body weight or food intake in rolipram-treated mice (Figure 2A and 2B), which suggests the anti-obesity effect of rolipram is independent of a change in food intake. We also found that the total cholesterol level decreased and adiponectin level increased in the livers of treated presenile mice as compared to untreated mice (Figure 2C and 2D). Based on these findings, we believe rolipram improves metabolic functions in presenile mice without significantly affecting body weight or food intake.

### AMPK is activated by increased cAMP levels in an animal model

**Figure 3:**
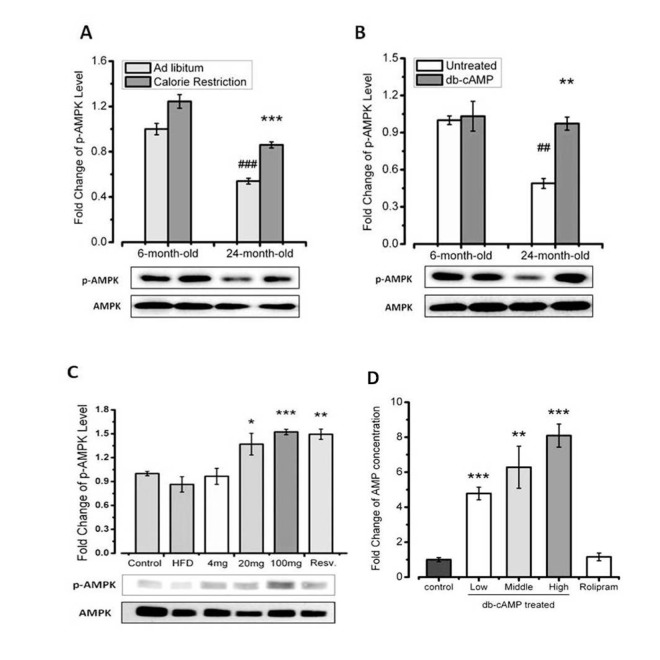
**AMPK is activated by increased cAMP levels in aged mice.** (**A**) Western blots showing the effct of AMPK in young and aged mice, with or without calorie restriction. (**B**) Western blots showing the effect of AMPK in young and aged mice, with or without db-cAMP treatment. (**C**) Western blots showing the effect of AMPK in HFD-fed mice treated with the indicated level of db-cAMP. (**D**) Hepatic AMP concentrations in mice treated with the indicated levels of db-cAMP or rolipram.

To investigate the mechanism underlying the beneficial effects of rolipram, we examined the effects of rolipram on cAMP levels in young (6 months old) and aged mice (21 months old) mice under several conditions. We also assessed phosphorylation of AMPK, which is an important energy sensor associated with aging and metabolism. We found that the increase in phosphor-AMPK elicited by db-cAMP administration is similar to that elicited by calorie restriction (Figure 3A and 3B). Our data also showed that db-cAMP induced dose-dependent increases in phosphor-AMPK in HFD-fed mice (Figure 3C).

We also observed that rolipram increased cAMP levels. When we then assessed the effect of rolipram on cAMP turnover by measuring levels of its metabolite, AMP, we found that rolipram increased cAMP levels without increasing levels of AMP. It thus appears that rolipram supports higher cAMP levels by reducing its turnover (Figure 3D). Taken together, these data indicate that rolipram administration leads to AMPK activition by increasing the cAMP concentration through inhibition of cAMP degradation to AMP.

### Rolipram increases the AMPK phosphorylation via CaMKKβ

**Figure 4:**
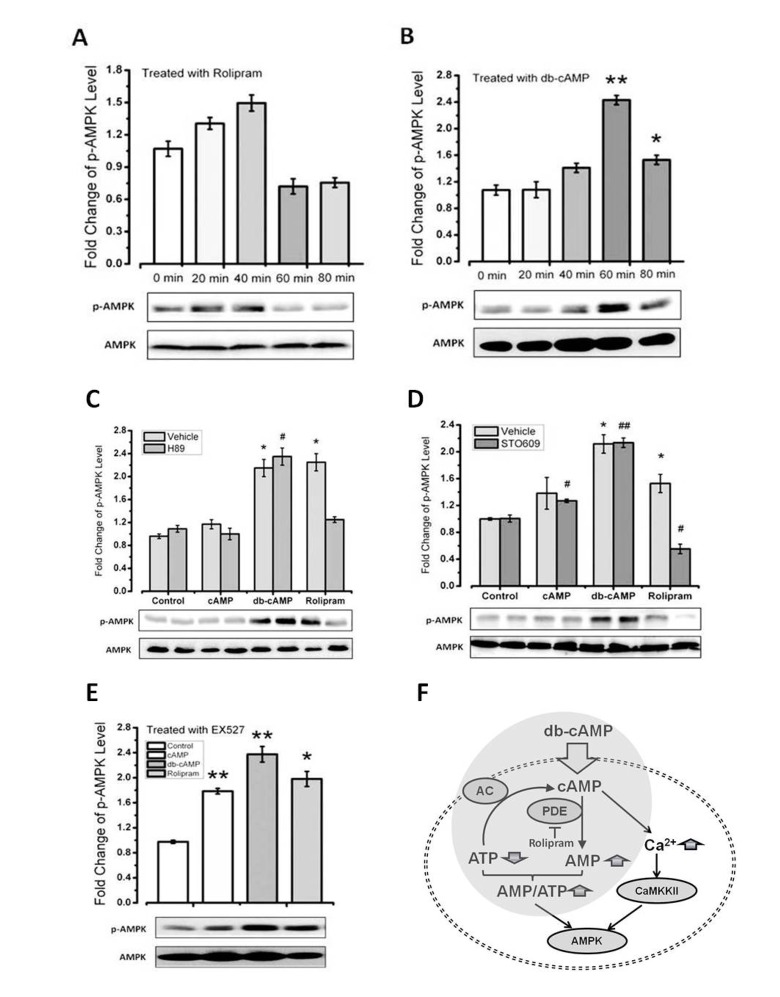
**Rolipram increases level of phosphor-AMPK via CaMKKβ**. (**A**-**B**) Time courses of the effects of db-cAMP and rolipram on phospho-AMPK levels in C2C12 cells. (**C**-**D**) Phospho-AMPK levels in cells treated with a PKA or CaMKKβ inhibitor. (**E**-**F**) Phospho-AMPK levels in cells treated with a SIRT1 inhibitor or expression SIRT6 siRNA.

Using the C2C12 cell line, we found that rolipram induced AMPK activation more rapidly than db-cAMP (Figure 4A and 4B). To determine whether db-cAMP mediates direct activation/phosphorylation of AMPK catalyzed by protein kinase A (PKA), we treated cells with the PKA inhibitor H89 or the CaMKKβ inhibitor STO609. We found rolipram-induced increases in phospho-AMPK were inhibited by STO609 (Figure 4C and 4D), which is consistent with an earlier observation [37]. By contrast, PKA inhibition had no effect on phospho-AMPK levels activation effect of cAMP levels, with or without CaMKKβ inhibition, suggesting that the mechanism by which rolipram mediates AMPK activation differs from the mechanism of cAMP.

PKA has been shown to rapidly activate SIRT1 [38]. In addition, we previously showed that treatment with db-cAMP leads to increase levels of SIRT1 protein [39]. Because AMPK activation is reportedly up-regulated via the SIRT1 pathway, we examined the effect of SIRT1 inhibition with EX527 on levels of phosphor-AMPK. We found that despite SIRT1 inhibition, both rolipram and db-cAMP increased phosphor-AMPK levels (Figure 4E). Similarly, because AMPK is reportedly activated via the SIRT6 pathway, we tested the effect of knocking down SIRT6 expression using SIRT6 siRNA on phosphor-AMPK levels. However, like SIRT1 inhibition, SIRT6 suppression had no effect on AMPK phosphorylation induced by db-cAMP or rolipram (Figure 4F). The rolipram-induced up-regulation of AMPK activation is thus independent of SIRT1 and SIRT6.

### Rolipram increases SIRT6 levels

**Figure 5:**
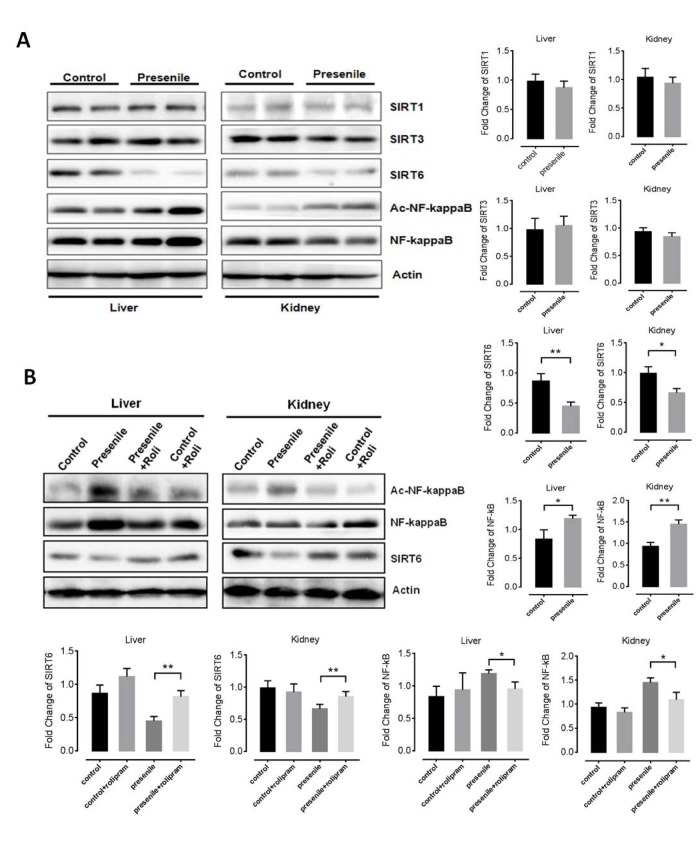
**Rolipram increases levels of SIRT6.** (**A**) Western blots results for SIRT1, SIRT3, SIRT6 and acetylated NF-κB in the livers and kidneys of presenile mice. (**B**) Western blot results for SIRT1, SIRT3, SIRT6 and acetylated NF-κB in the livers and kidneys of presenile mice, with or without rolipram treatment.

As cross-talk between sirtuin family proteins and the AMPK pathway plays a key role in aging and metabolism, we investigated the relationship between sirtuin family proteins (SIRT1, SIRT3 and SIRT6) and premature aging-like phenotypes in presenile mice. Interestingly, SIRT1 and SIRT3 levels did not significantly differ between normal and presenile mice. By contrast, SIRT6 levels were lower in the kidney and livers of presenile mice than normal mice (Figure 5A). Because computational genomics analyses have shown increased NF-κB activity in multiple *Sirt6*-deficient tissues *in vivo* [31], we also assessed NF-κB acetylation. We found NF-κB acetylation to be increased in the livers and kidneys of presenile mice, but that effect was reversed by rolipram (Figure 5B). These results suggest the anti-aging effect of rolipram may be dependent on the up-regulation of SIRT6 signaling and inhibition of the NF-κB pathway downstream of up-regulated AMPK activity.

### Rolipram decreases NF-kB acetylation

**Figure 6:**
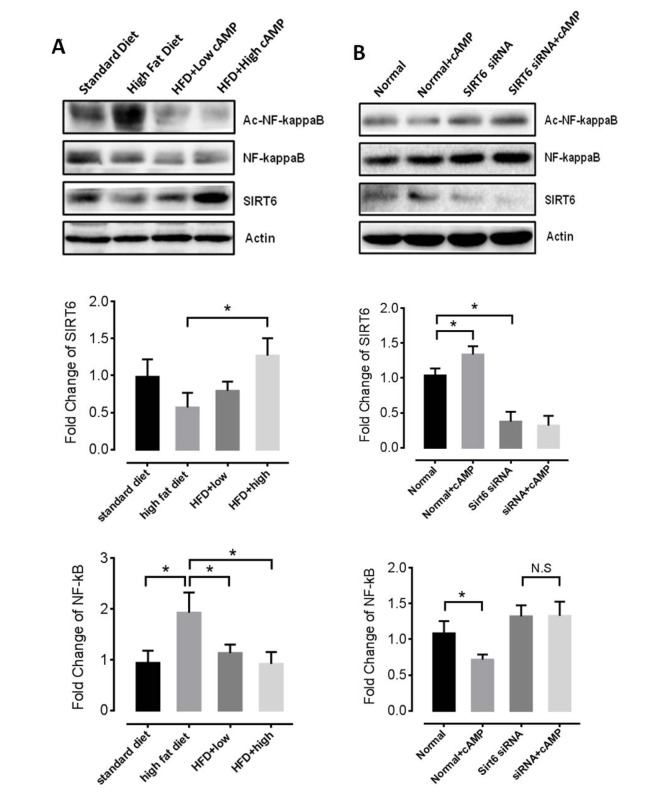
**Roliparm decreases levels of acetylated NF-**κ**B.** (**A**) SIRT6 protein and NF−κB acetylation level of HFD-fed mice with or without db-cAMP treatment. (**B**) SIRT6 and acetylated NF-κB levels in *Sirt6* knockdown and control cells, with or without db-cAMP treatment.

Because rolipram increases cAMP levels by inhibiting its cAMP hydrolysis, we treated mice with db-cAMP to investigate the mechanism of the anti-aging effect of rolipram. We found that db-cAMP increased SIRT6 levels and decreased the NF-κB acetylation in the livers of HFD-fed mice (Figure 6A), which is consistent with an earlier report [33]. We also tested mRNA levels of IL-1, IL-6 and TNFα with or without rolipram treatment in cultured cell and found that IL-1 level decreased while TNFα level increased when SIRT6 siRNA was applied (Figure S2). Moreover, in cells where SIRT6 expression was knocked down using SIRT6 siRNA, rolipram no longer inhibited NF-κB acetylation (Figure 6B), suggesting rolipram acts via the SIRT6 pathway to suppress NF-κB acetylation.

## DISCUSSION

**Figure 7:**
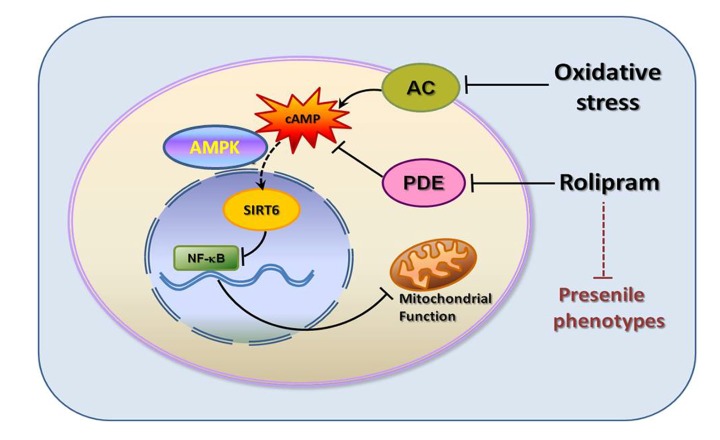
Graphic summary.

Although much has been learned about the potential benefits of PDE4 inhibitors in the treatment of aging-related diseases such as Alzheimer's disease [7,8,19], relatively little is known about their effects on adipose deposition and metabolic disorders during aging. Our present investigation demonstrated for the first time that the PDE4 inhibitor rolipram as well as a cAMP analogue suppressed senescence-associated adipose deposition. Rolipram increased the cAMP concentration and protected subcutaneous adipose while decreasing adipose deposition in the livers and kidneys of presenile mice. The rolipram-incuced increases in cAMP also led to AMPK activation, which in turn led to up-regulation of SIRT6 and suppression of aging-associated phenotypes. Further experiments showed that NF-κB acetylation was increased when SIRT6 levels were decreased. Our data thus reveal that rolipram administration leads to higher cAMP concentrations, activation of the energy sensor AMPK, and up-regulation of SIRT6, a key cascade involved in aging and metabolism (Figure 7).

In patients with aging-related diseases, such as diabetes and cardiovascular disease, adipose deposition in organs and vascular system is often a major cause of chronic inflammation that underlies pathophysiology and functional decline [1,36]. In this case, rolipram-mediated prevention age-related adipose deposition and metabolic disorder would be beneficial for multiple senescence-associated diseases.

In recent years, there has been increasing interest in screening drugs for metabolic disorders. One anti-aging drug candidate, resveratrol, reportedly increases the intracellular cAMP concentrations in a manner similar to rolipram [22]. Here, we observed that rolipram increases AMPK phosphorylation via CaMKKβ. AMPK activation reportedly raises NAD^+^ concentrations, which may in turn increase the activity of sirtuin family proteins [27]. In our study, hepatic and renal SIRT6 levels were significantly reduced in presenile mice, but levels were restored by rolipram. We previously reported that cAMP stabilized sirtuin family proteins by protecting the NAD^+^ binding site [39]. We speculate that rolipram mediates up-regulation of SIRT6 based through two mechanisms: 1) rolipram increases the cAMP concentration, which stabilizes the SIRT6 at a relatively high level; and 2) cAMP activates AMPK, which induces NAD^+^ production and enhances SIRT6 activity.

Among candidate anti-aging drugs, rolipram shows considerable potential because it has already been used clinically to treat depression treatment, and there is a clear background of pharmacokinetic and toxicological information. Moreover, rolipram has already been shown to improve learning and memory functions in mice with Alzheimer's disease. Our results showed that rolipram administration can improve aging-associated adipose distribution in presenile mice. Further investigation of the molecular mechanism showed that age-associated adipose deposition was regulated via the cAMP-AMPK-SIRT6 pathway, and that rolipram improves adipose metabolism.

## MATERIALS AND METHODS

### Animals and diets

Young (3-month-old) male BABL/c mice were purchased from Vital River Laboratories (Charles River Laboratories) and treated according to guidelines for the care and use of laboratory animals and with the approval of the Institutional Ethical Committee of China. Eight-week-old male mice were housed and maintained on a 12 h light-dark cycle (light on 8 am–8 pm) with free access to food and water for the first month, until they reached 12 weeks of age. For all db-cAMP-related studies, mice were orally administered 4, 20, or 100 mg/kg db-cAMP (in food) or with 100 mg/kg resveratrol as a positive control. HFD-fed mice were fed a diet containing 40% of calories from fat (AIN-93, Research Diets) for up to 12 weeks. To measure the effect of cAMP on calorie restriction, mice were subjected to limited food intake (60% of the daily food intake by weight) for 3 months, with free access to water. For studies involving aged mice treatment with cAMP, mice had free access to food and water from 12 weeks until death, n = 20 per group. However, 3-5 aged mice from each group were sacrificed for different experiments.

### Cell culture and treatment

3T3-L1 cell were cultured in Dulbecco’s modified Eagle medium (DMEM) (Thermo Scientific) supplemented with 10% fetal bovine serum (FBS) in 5% CO_2_. Rolipram (Enzo Life Science). db-cAMP was added to cells for 1 h before harvesting. Cells were seeded in culture plates (Corning) at a density of 2–3 x10^4^ cells/well and incubated in high-glucose DMEM supplemented with 10% FBS and 1% penicillin/streptomycin. On the day of testing, cells were incubated for 6 h in fresh DMEM containing 10, 100, 1000 mM db-cAMP, 100 mM rolipram or DMSO (vehicle control) with or without the PKA inhibitor H89, the CaMKKβ inhibitor STO609 or the AMPK inhibitor Compound C. The cells were then washed twice with 5 mL of assay medium (unbuffered low glucose DMEM supplemented with pyruvate and glutamine, pH 7.4).

### ROS measurements

ROS levels were determined in muscle extracts using the ROS-sensitive fluorescent dye dichlorodihydrofluorescein (DCF). The oxidation-insensitive dye carboxy-DCFDA was used as a control to ensure that changes in the fluorescence seen with the oxidation-sensitive dye H2DCFDA were due to changes in ROS production. Both dyes were first dissolved at a concentration of 12.5 mM and diluted with homogenization buffer to 125 mM immediately before use. Diluted dyes were added to tissue extract (100 mg) in a 96-well plate to achieve a final concentration of 25 mM. The change in fluorescence intensity was monitored at two time points (0 and 30 min) using a microplate fluorescence reader (Bio-Tek Instruments), with excitation set at 485 nm and emission set at 530 nm.

### Histochemical section observation

Liver and kidney adipose tissue section were stained by haematoxylin and eosin. Collagen in skin was stained by Masson’s Trichrome. The staining procedures followed the standard protocol. Observation made using a light microscope (Leica) equipped with 10x and 40x objectives.

### Western-blot analysis

Animal tissues were lysed and centrifuged, after which protein concentrations in the lysates were determined using the BCA method. Aliquots of tissue homogenates were mixed with loading buffer and heated. Samples for western blotting were resolved on 10% SDS-polyacrylamide gels and transferred to PVDF membranes. The following antibodies against the following proteins were used: SIRT1, SIRT3, total AMPK, phosphor-AMPK (Thr-172), NF-kB (all from Cell Signaling Technology); SIRT6 (Abgent) and actin (Santa Cruz, USA).

### RNA isolation and real-time PCR analysis

Total RNA was isolated using a Trizone and RNA purification kit (Tiangen, China) according to the manufacturer’s instructions. The obtained RNA was reverse transcribed into cDNA. Expression of mRNAs was assessed using real-time quantitative reverse transcription PCR using a SYBR Green PCR Mix kit. Levels of targeted mRNAs were normalized to the level of actin RNA.

### Serum parameters

After treatment, mice were sacrificed, and their blood was collected from the orbital venous plexus. Serum cholesterol and adiponectin was measured using commercially available ELISA kits according to the manufacturer’s instructions.

### Statistical analyses

All data were expressed as the mean ± SEM. Comparisons between groups were made using Student’s t test comparison or one-way ANOVA followed by Tukey’s multiple comparison. Differences were considered statistically signiﬁcant at p<0.05. Data analyses were performed using the statistical program Origin Graph.

## Supplementary Material

Supplementary Figure 1

Supplementary Figure 2

Supplementary Figures 3-6
