# Supplementary figures and images for: Phosphodiesterase 4 inhibitor activates AMPK-SIRT6 pathway to prevent aging-related adipose deposition induced by metabolic disorder
Source: Aging (Albany NY). 2018 Sep 18;10(9):2394–406. doi: 10.18632/aging.101559 (PMC6188481; doi:10.18632/aging.101559)

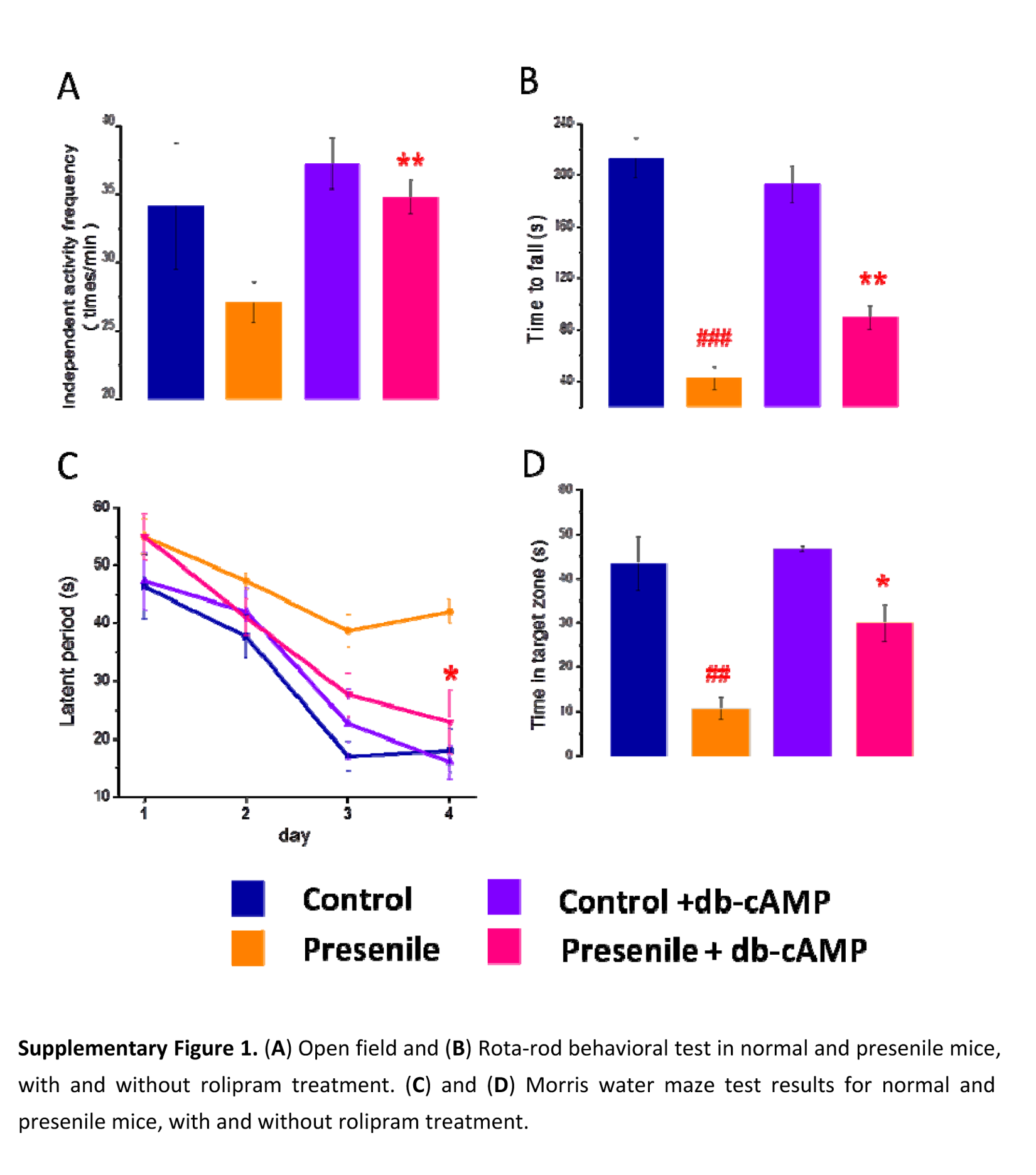

Supplement: Supplementary Figure 1 [file aging-10-101559-s001.tif]

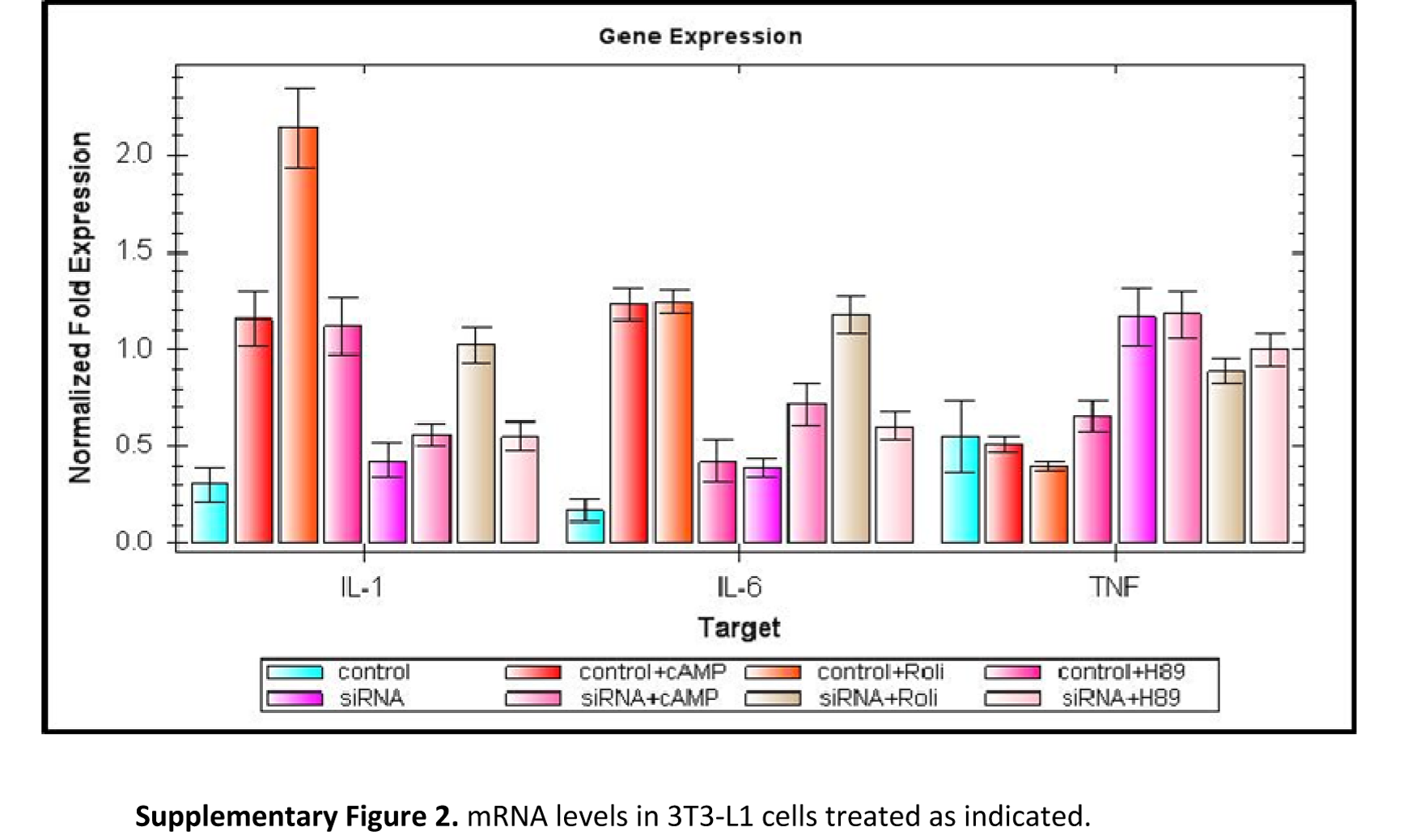

Supplement: Supplementary Figure 2 [file aging-10-101559-s002.tif]
