# Supplementary material for: Phosphodiesterase 4 inhibitor activates AMPK-SIRT6 pathway to prevent aging-related adipose deposition induced by metabolic disorder
Source: Aging (Albany NY). 2018 Sep 18;10(9):2394–406. doi: 10.18632/aging.101559 (PMC6188481; doi:10.18632/aging.101559)

# Figure 3A. p-AMPK

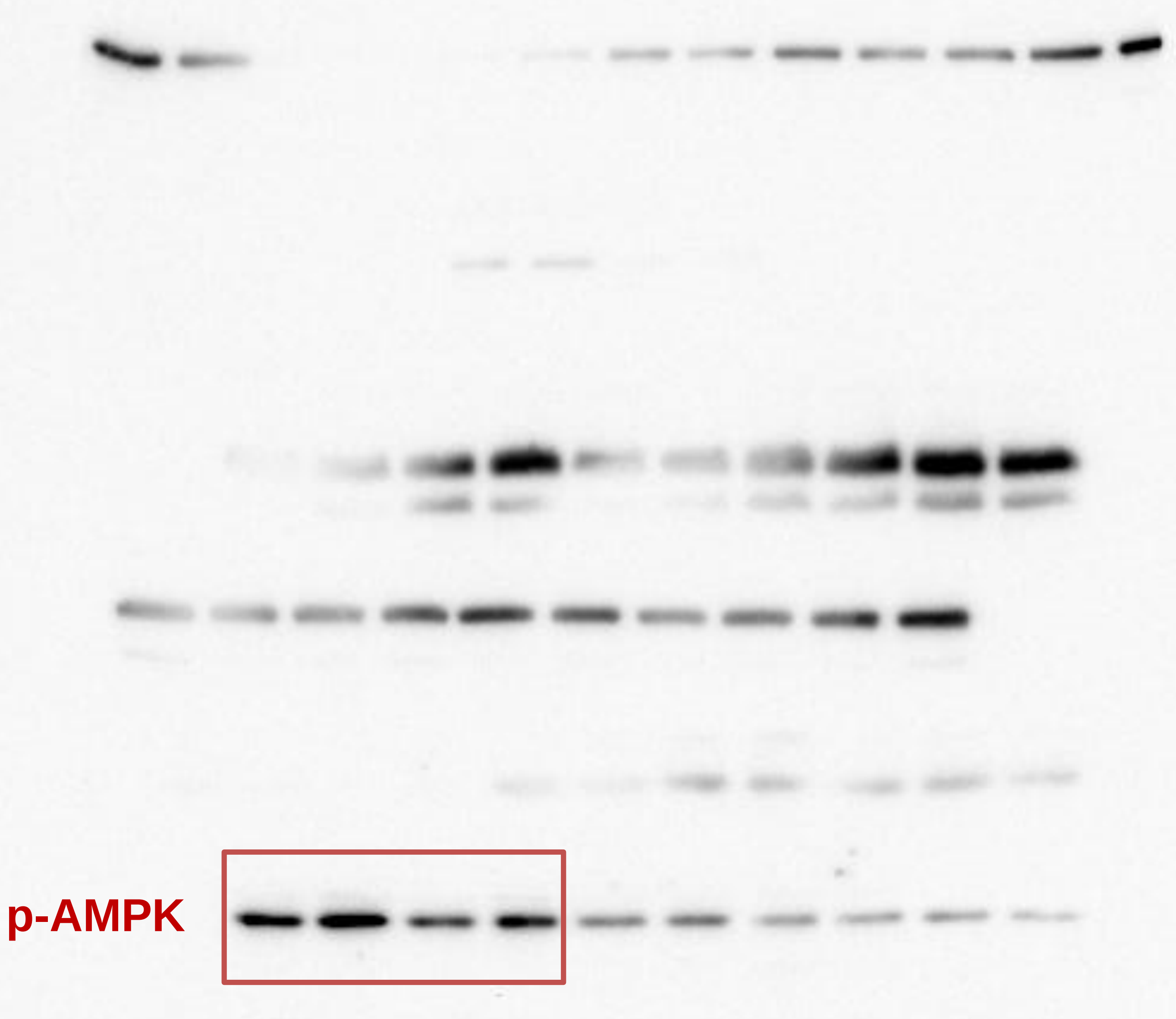

Figure 3A. AMPK

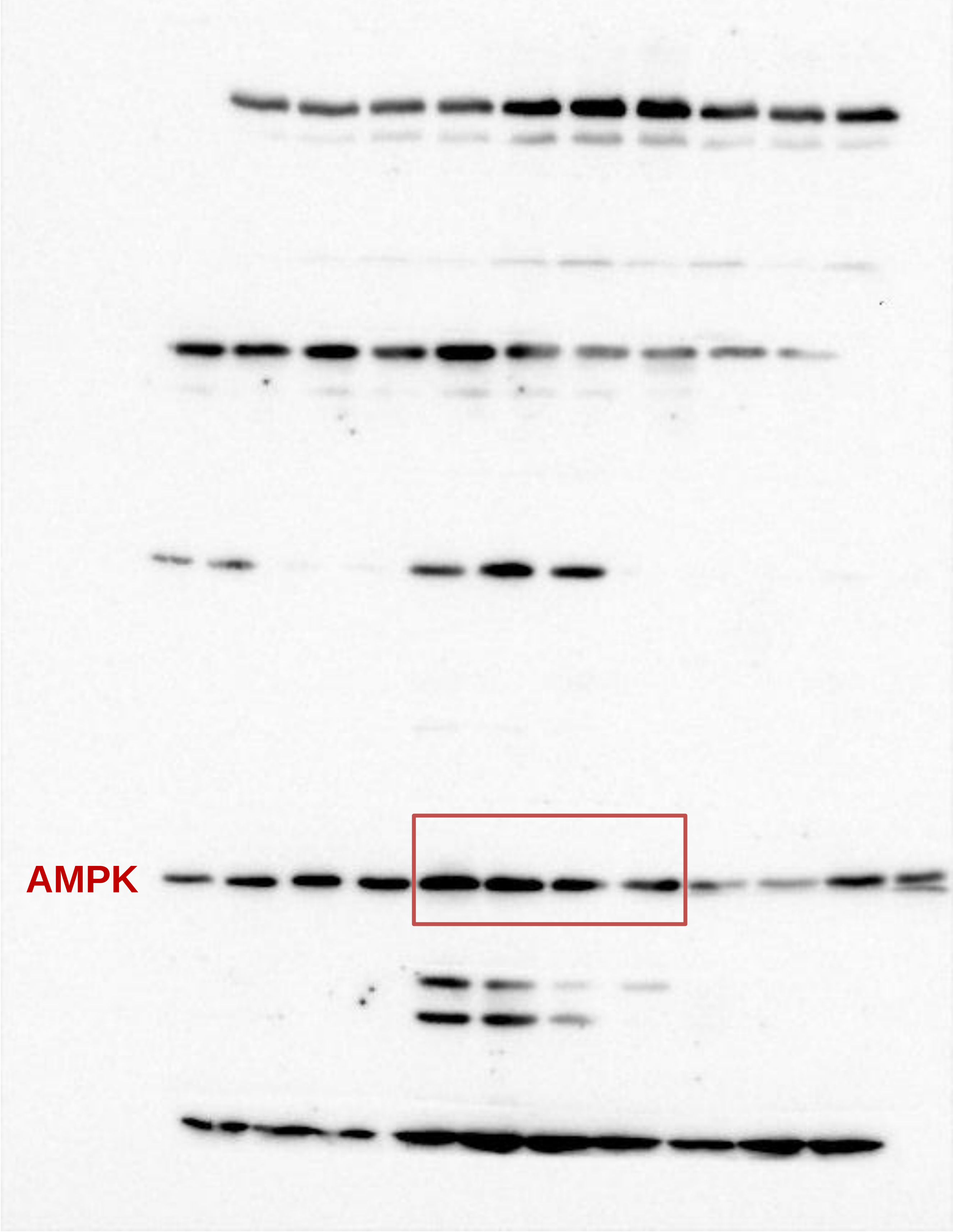

## Figure 3B. p-AMPK

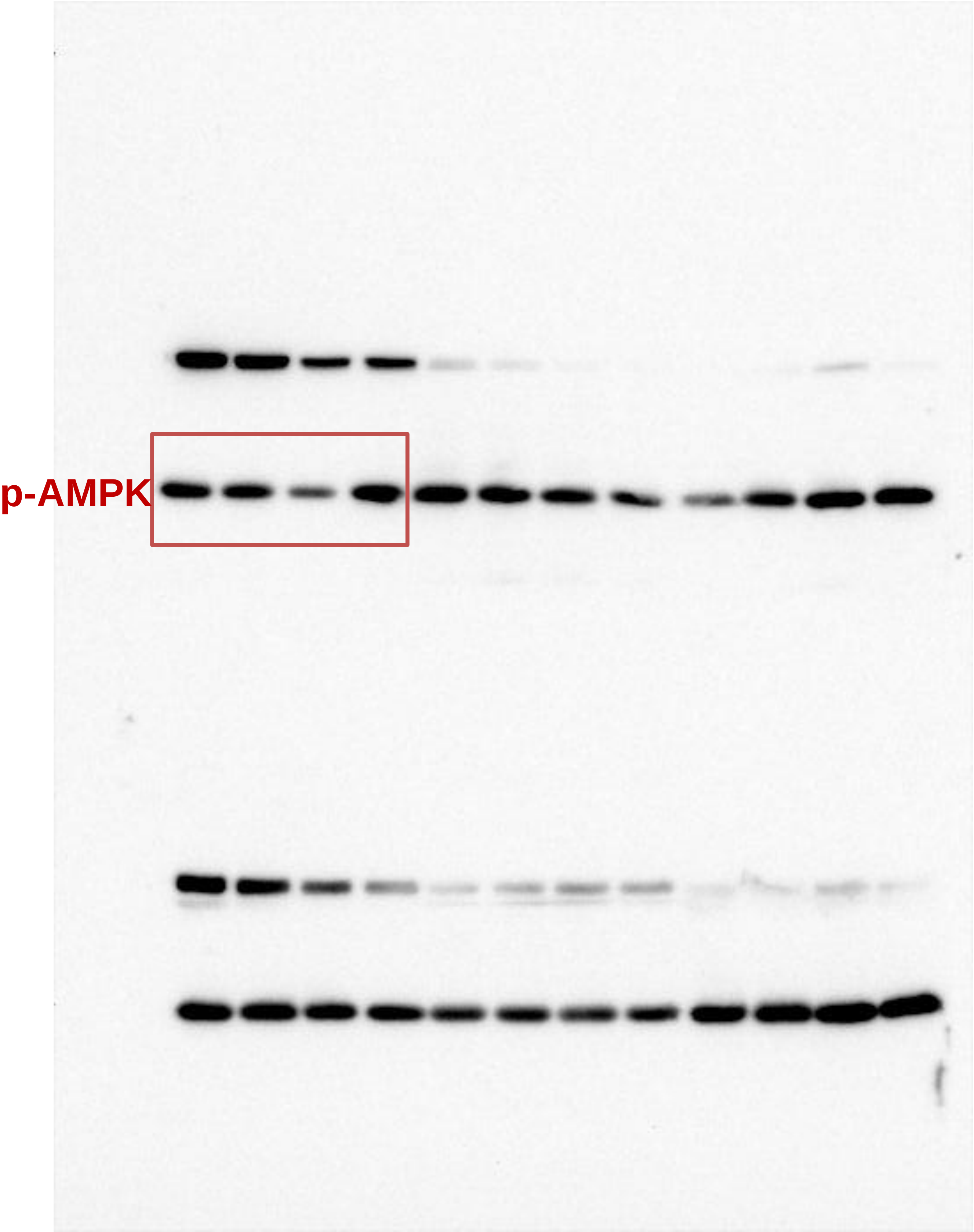

# Figure 3B. AMPK

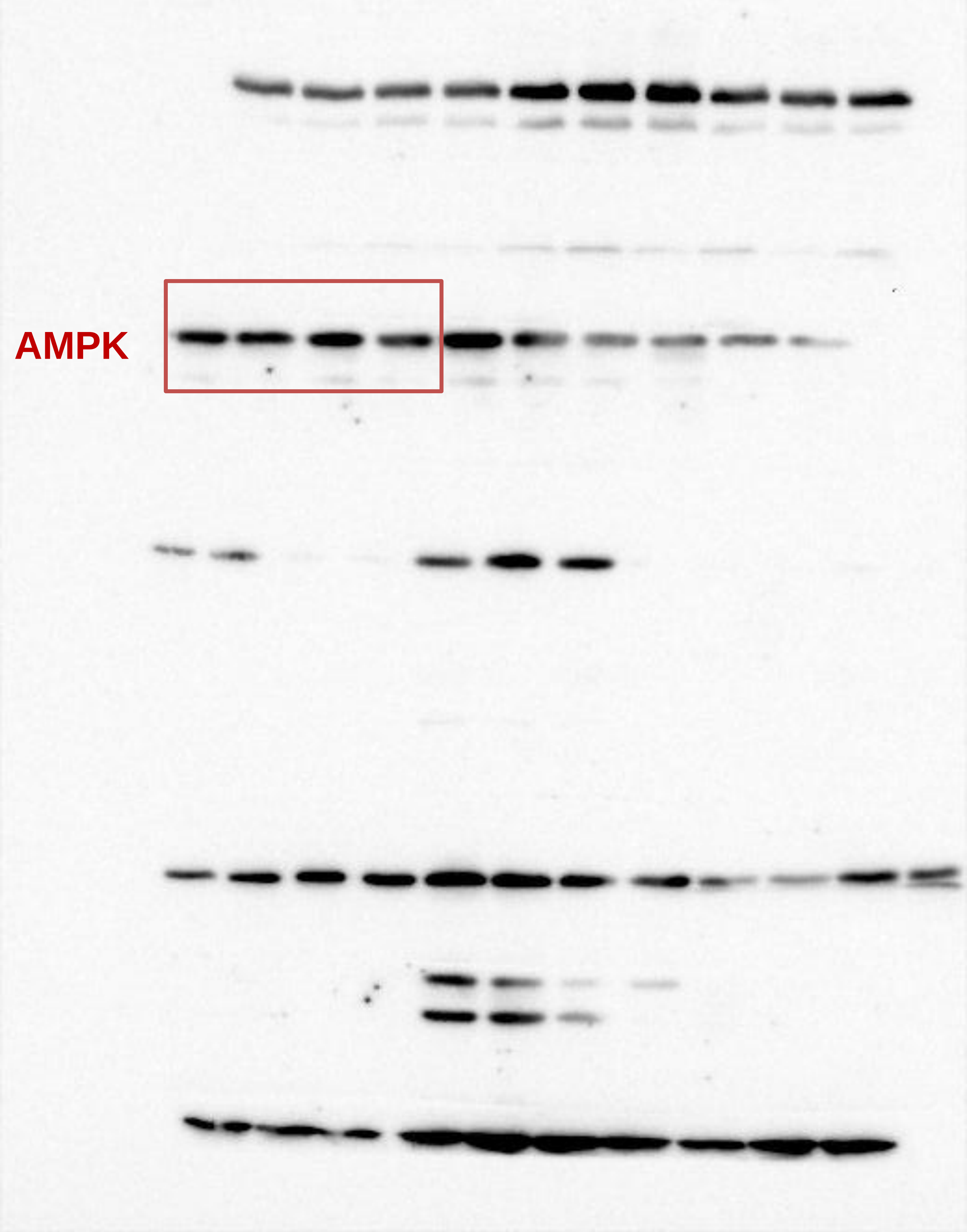

## Figure 3C. p-AMPK

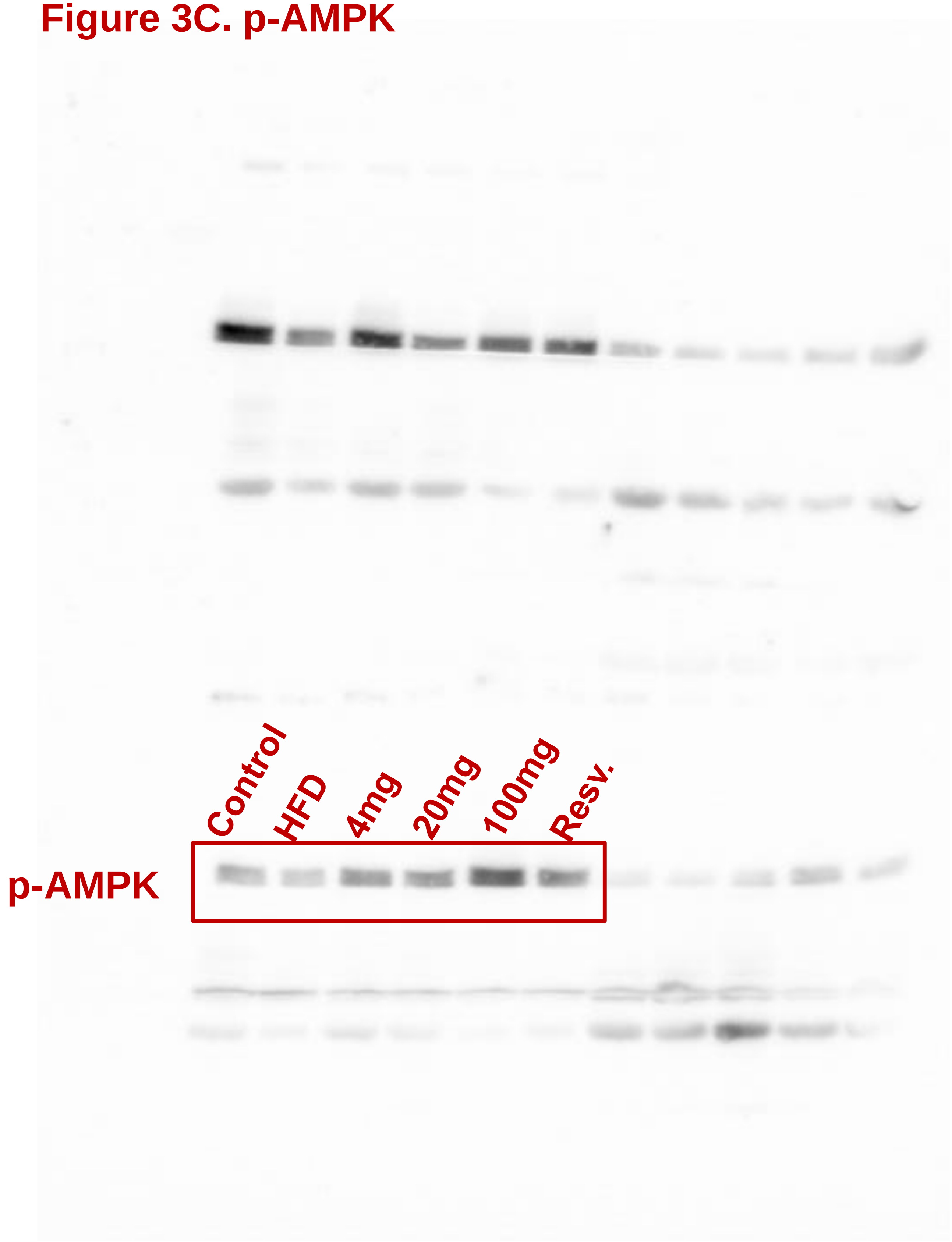

# Figure 3C. AMPK

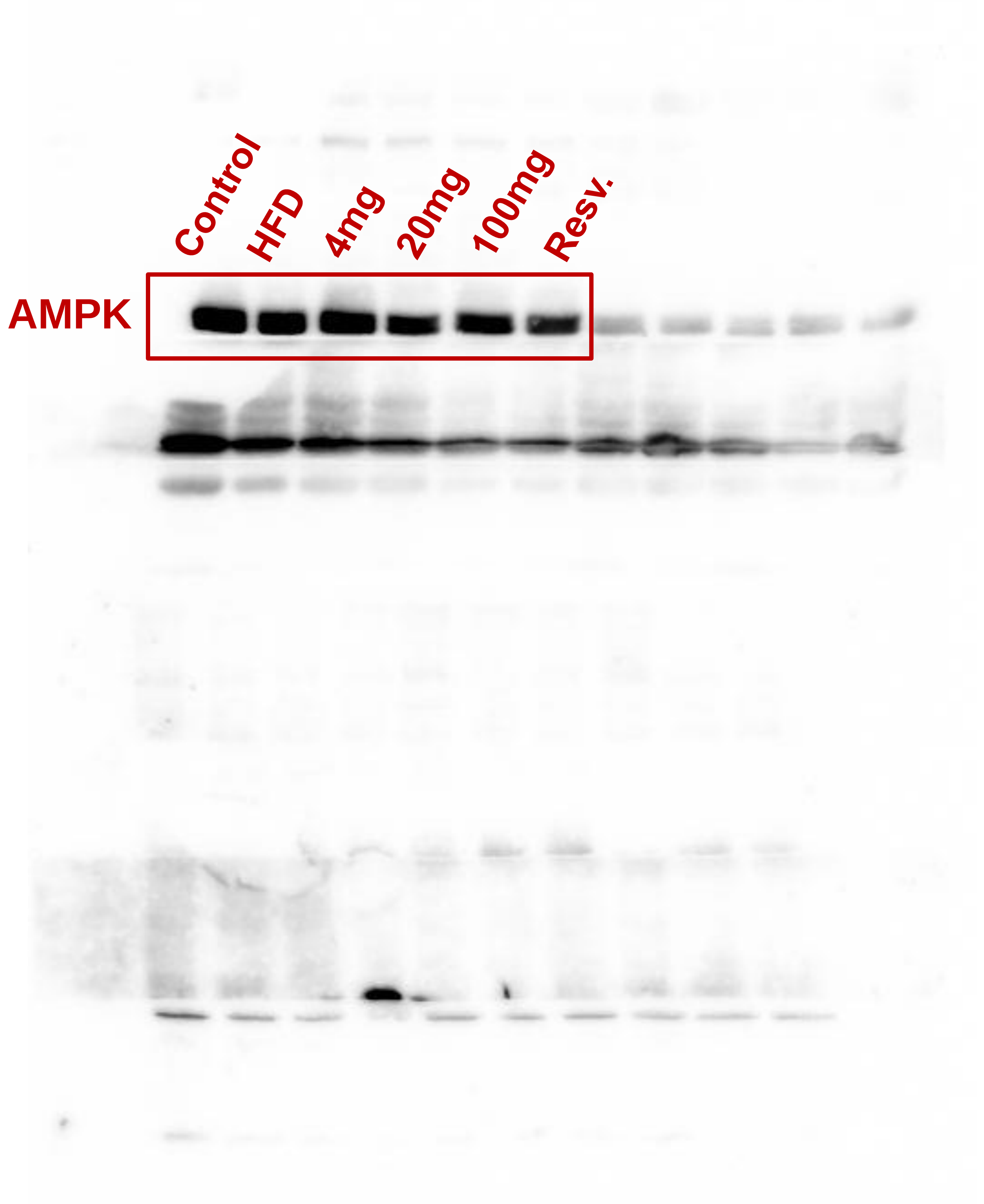

## Figure 4A. AMPK

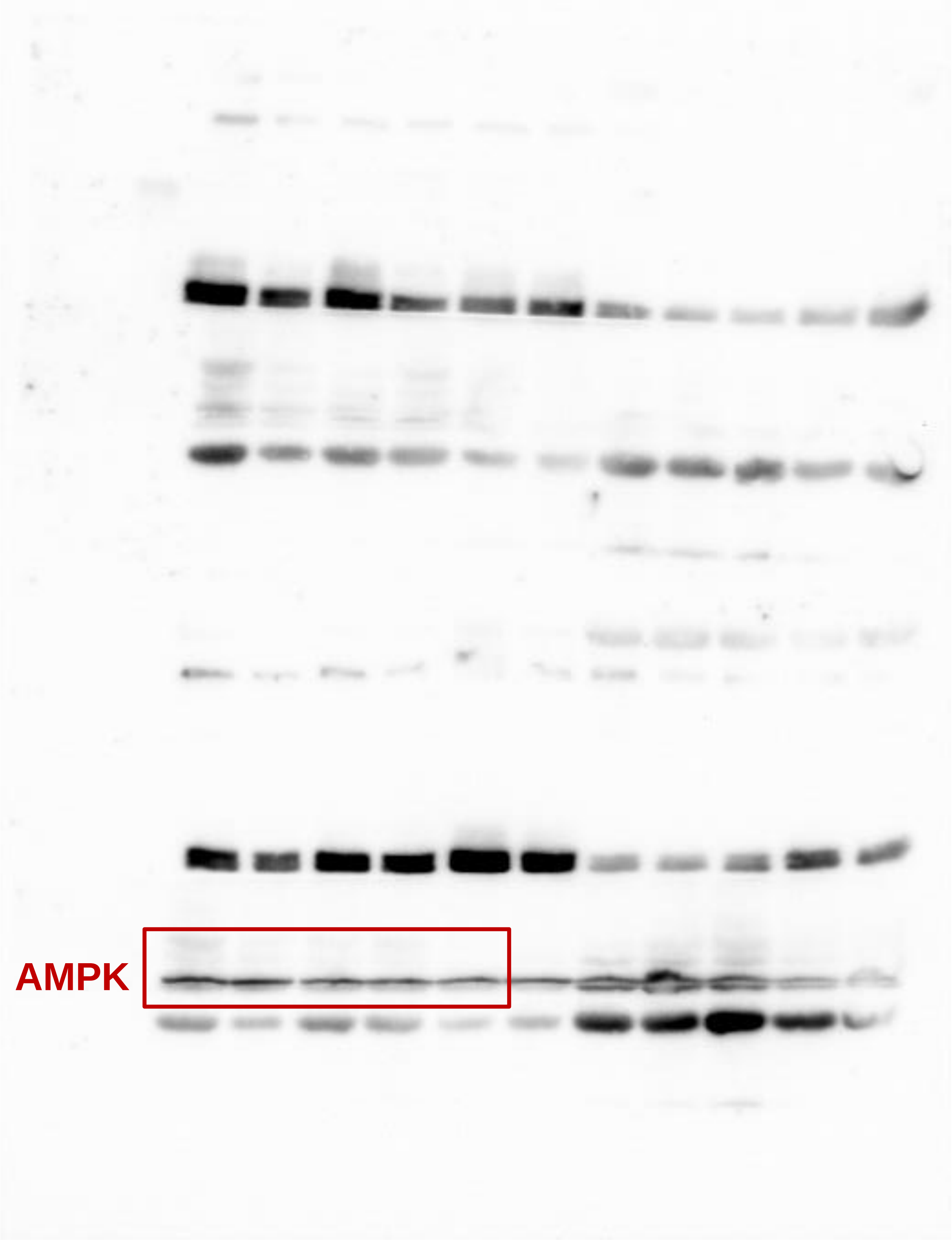

**Figure 4B. p-AMPK**

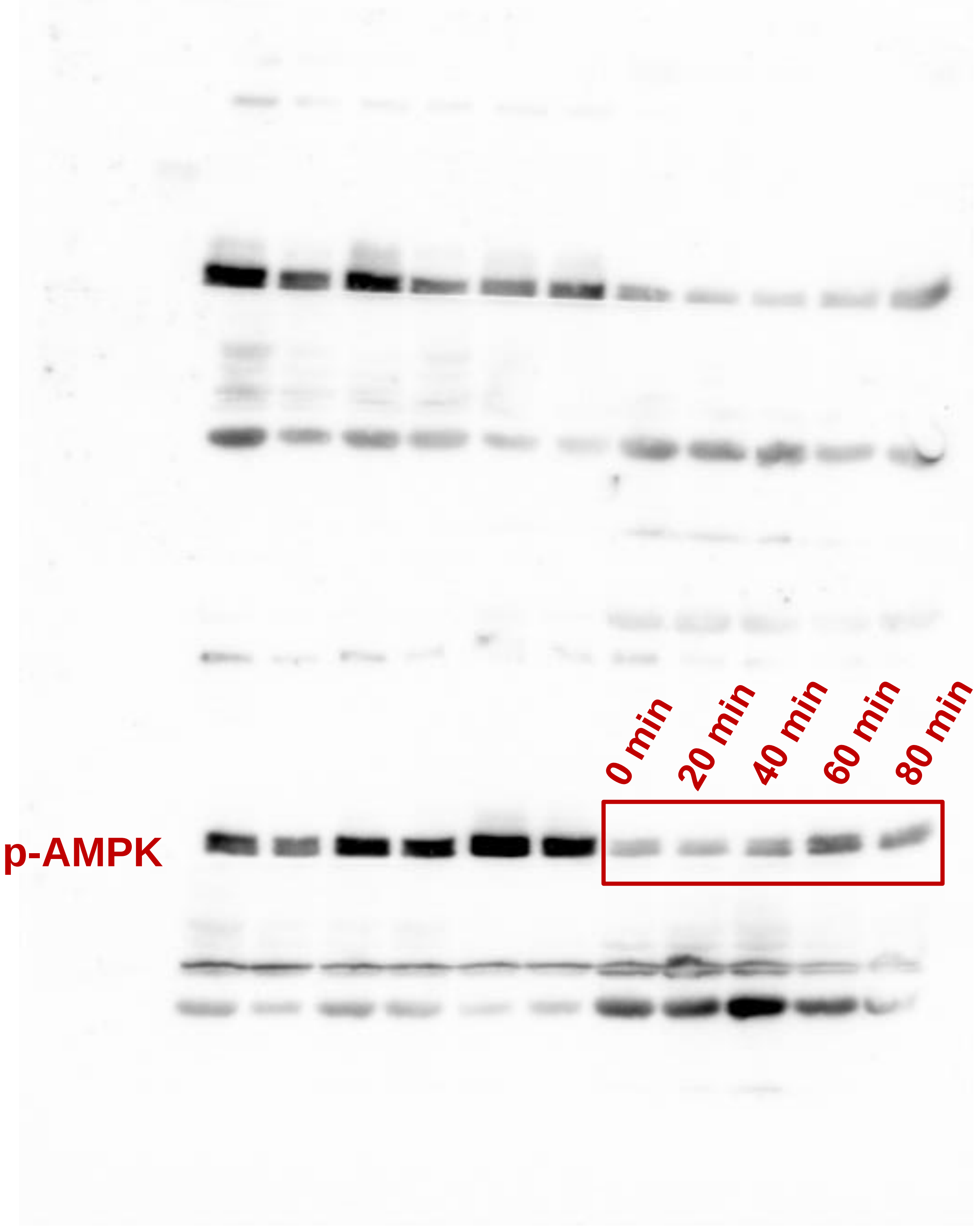

# Figure 4B.  AMPK

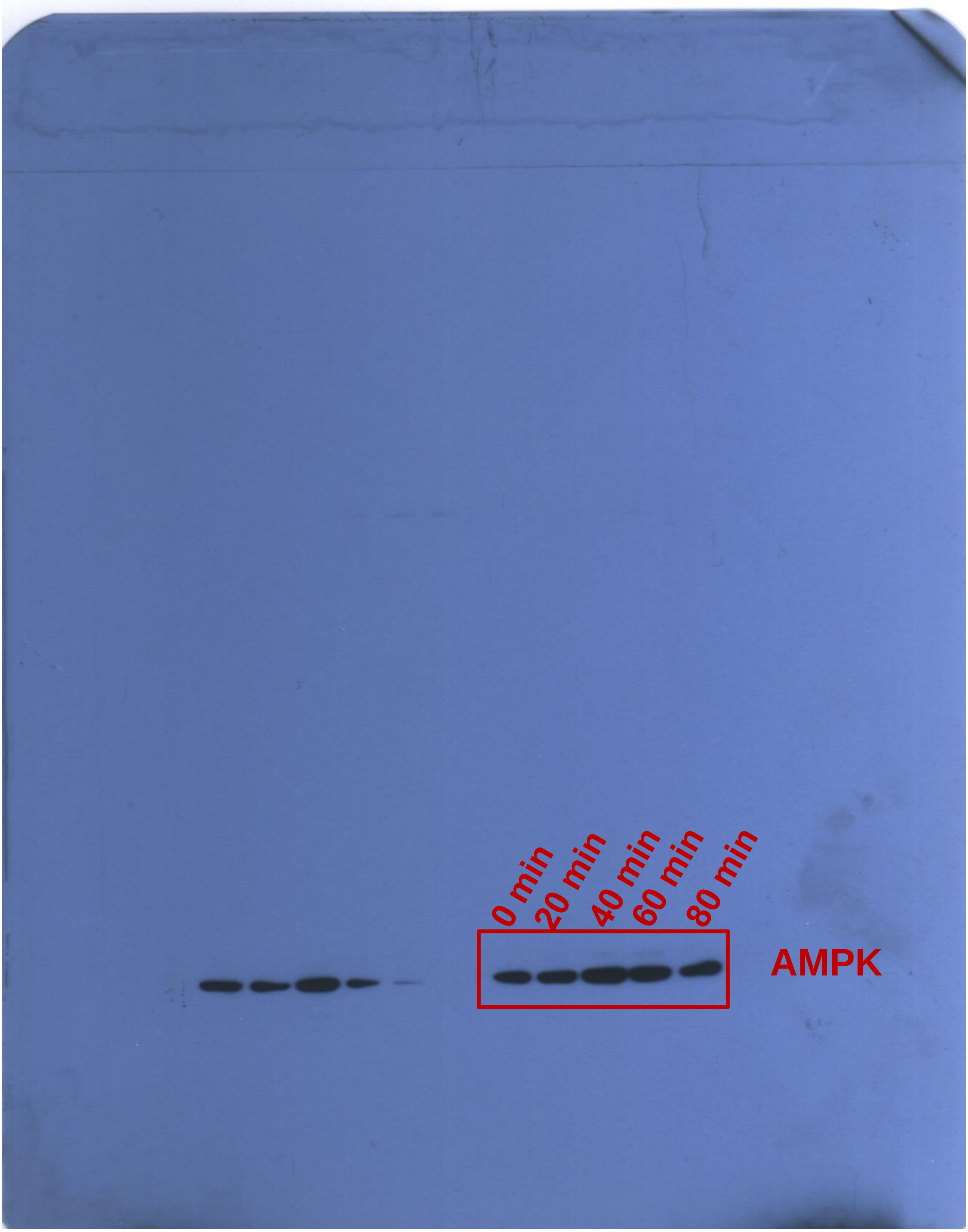

**Figure 4C. p-AMPK**

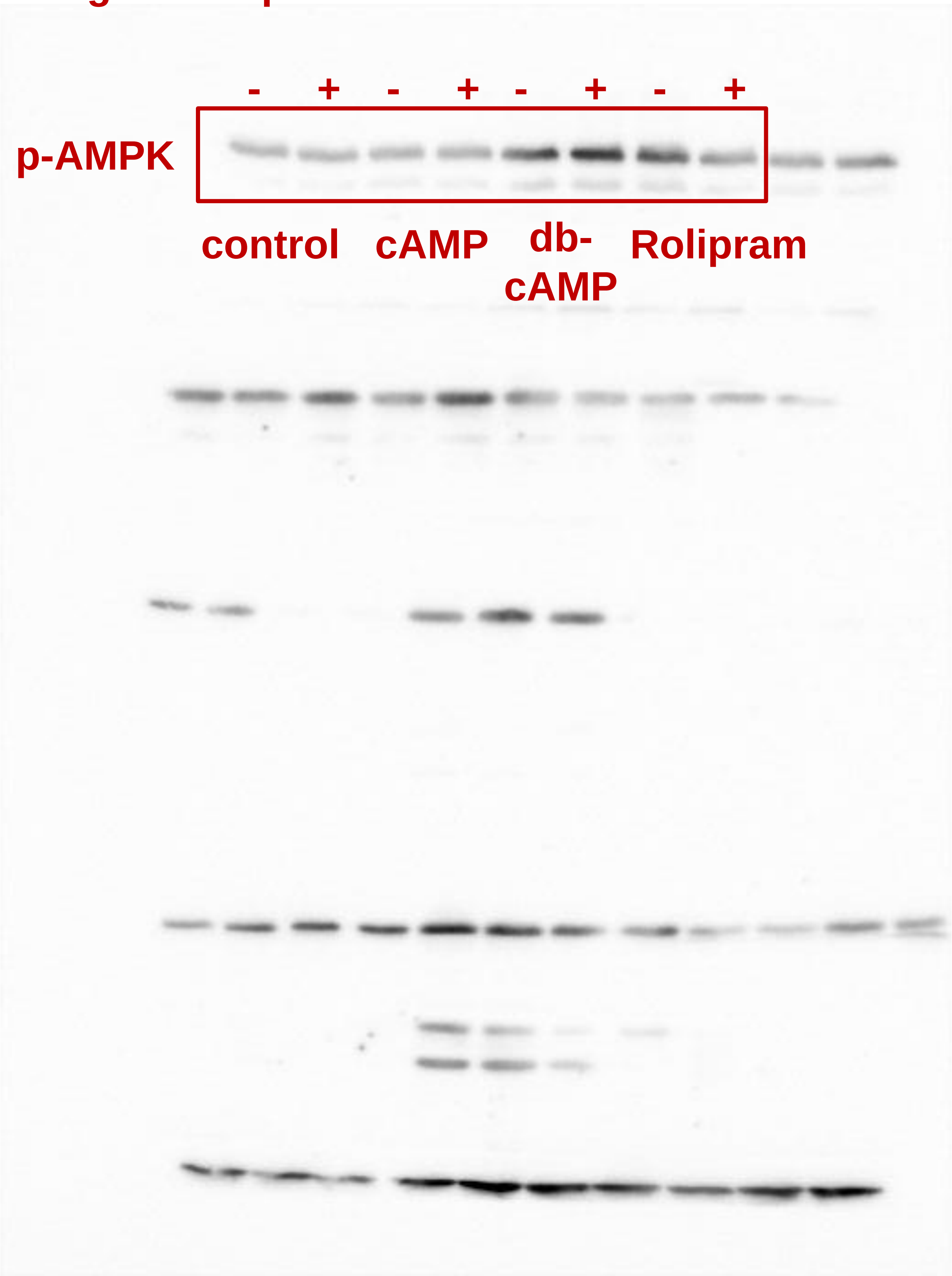

# Figure 4C. AMPK

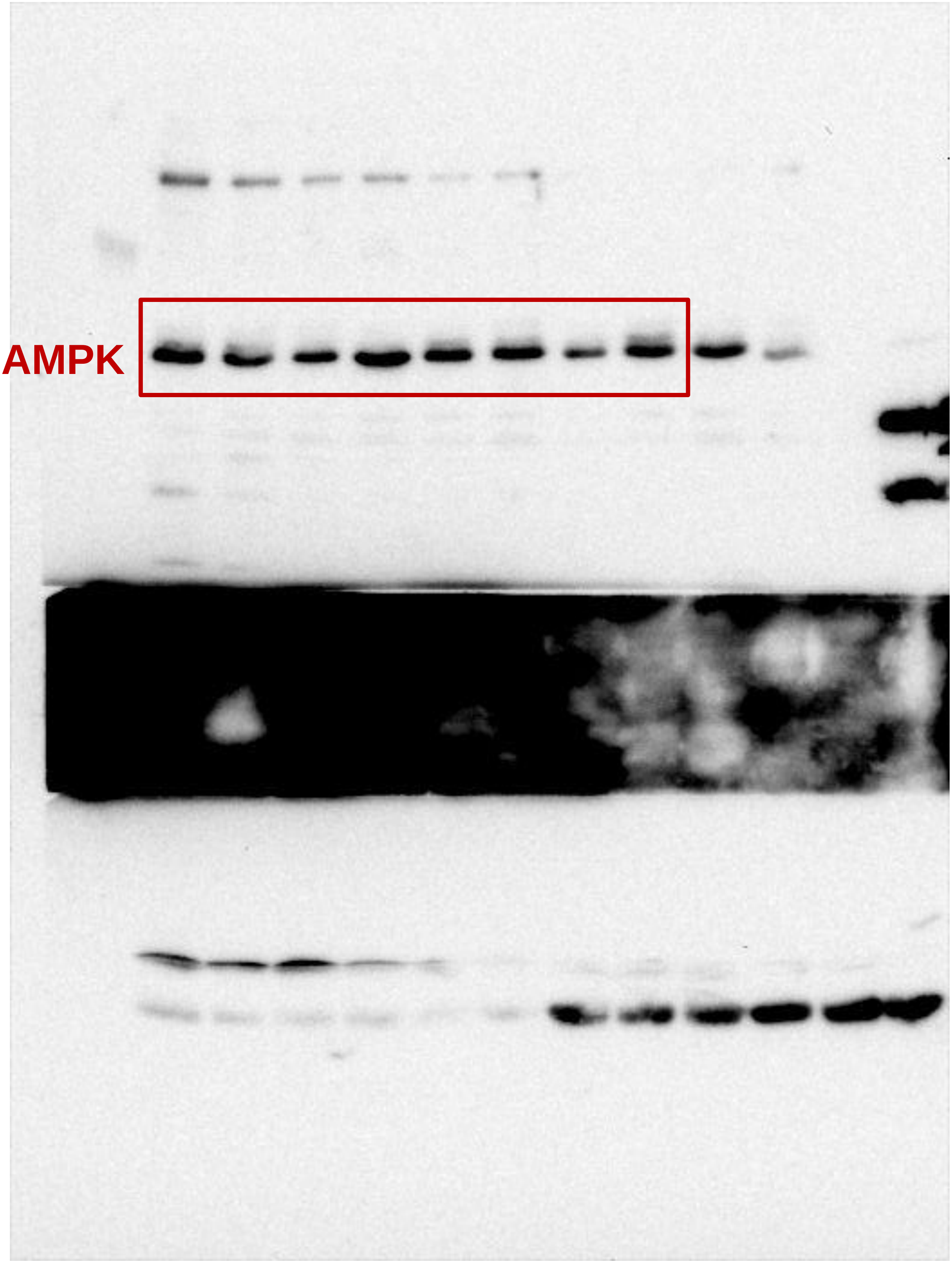

# Figure 4D. p-AMPK

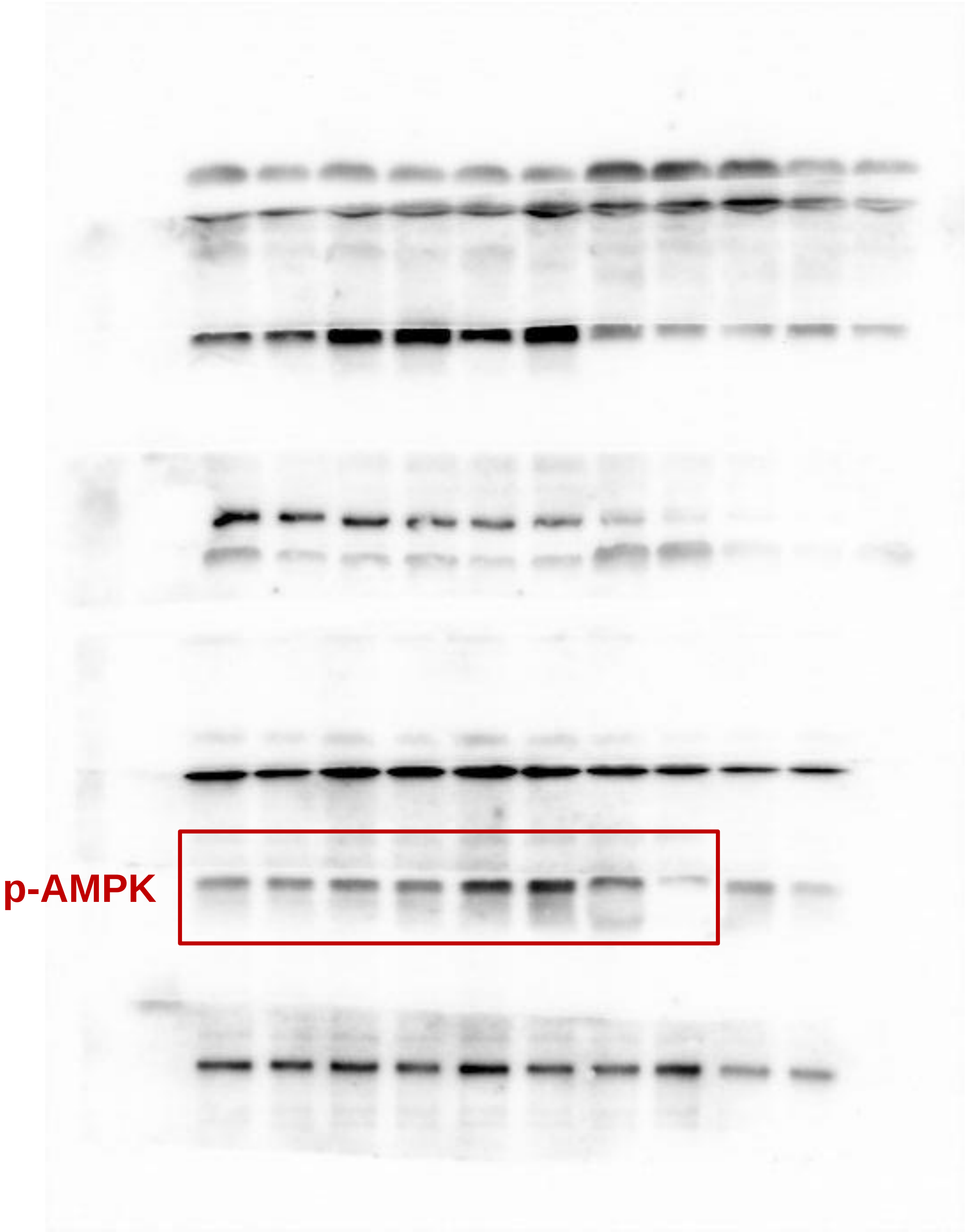

# Figure 4D. AMPK

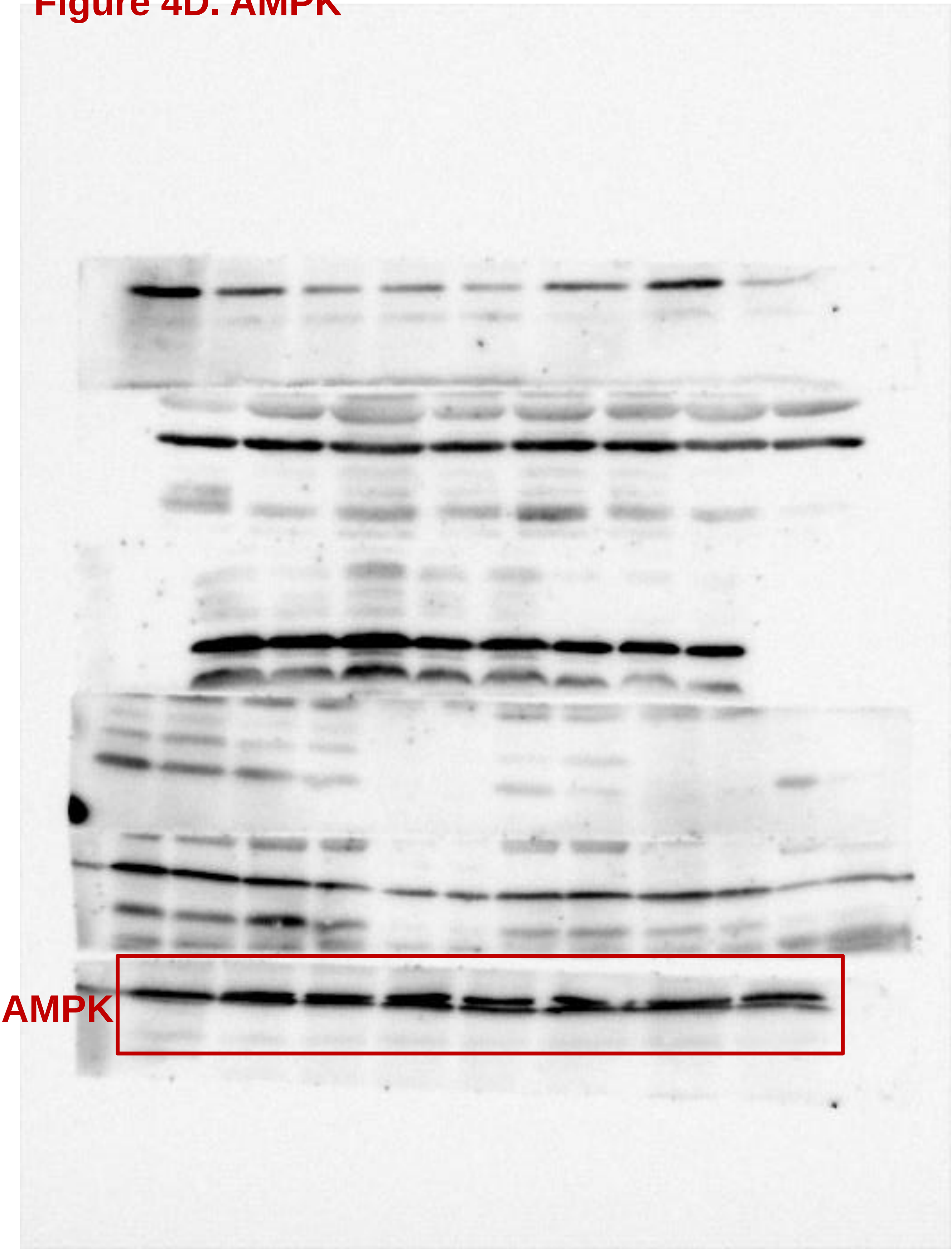

**Figure 4E. AMPK**

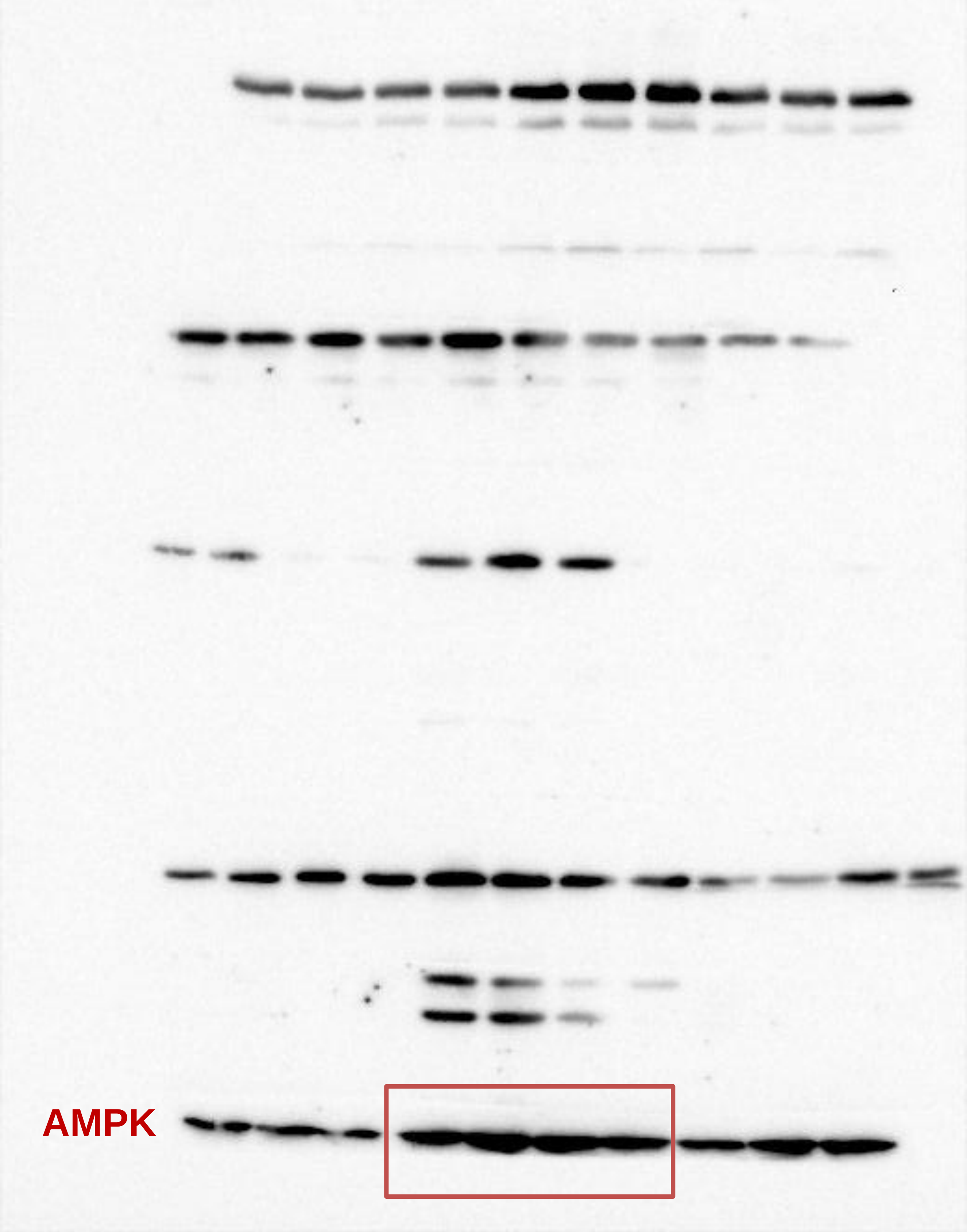

# Figure 5A. SIRT1

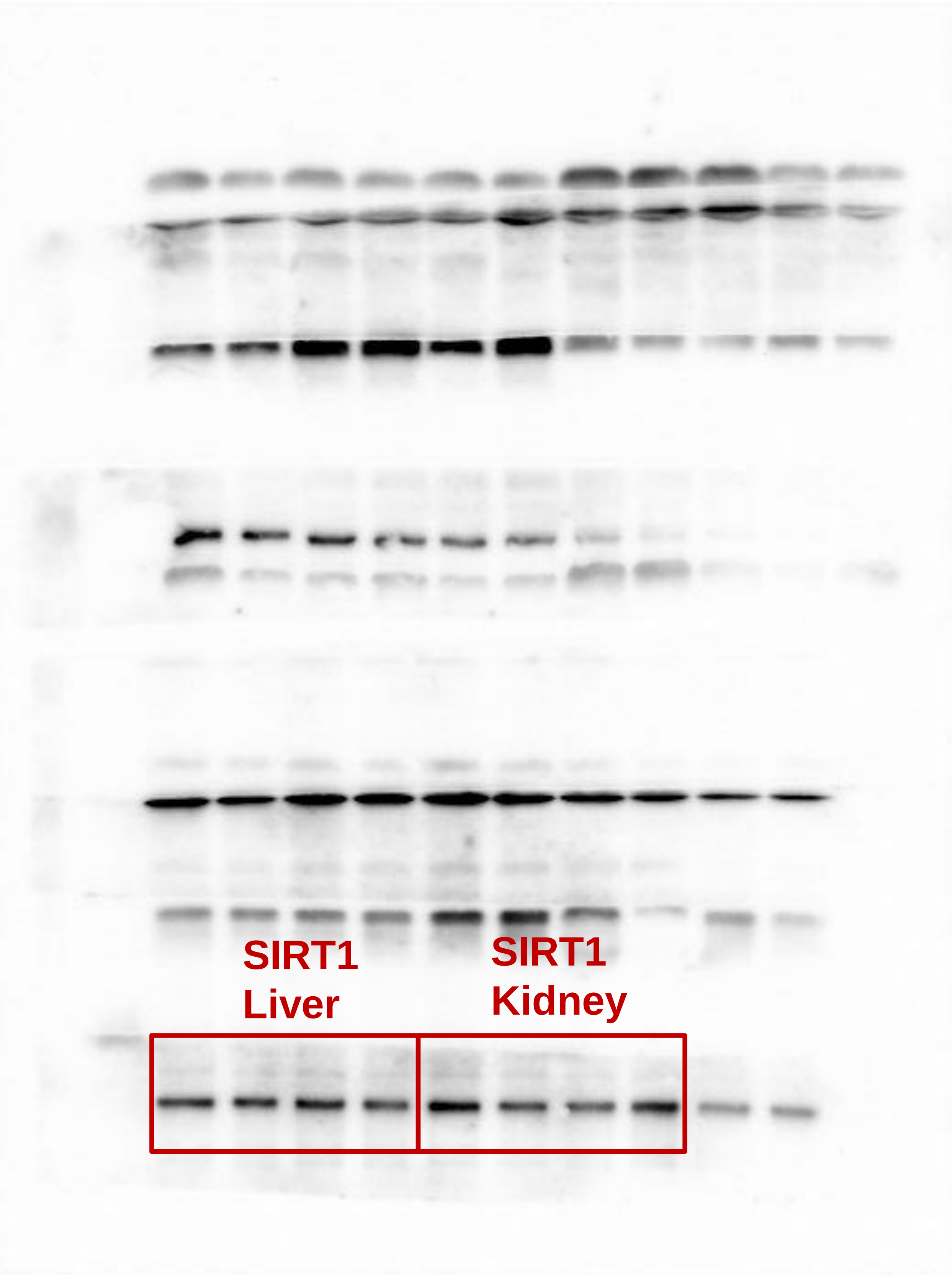

# Figure 5A. SIRT3

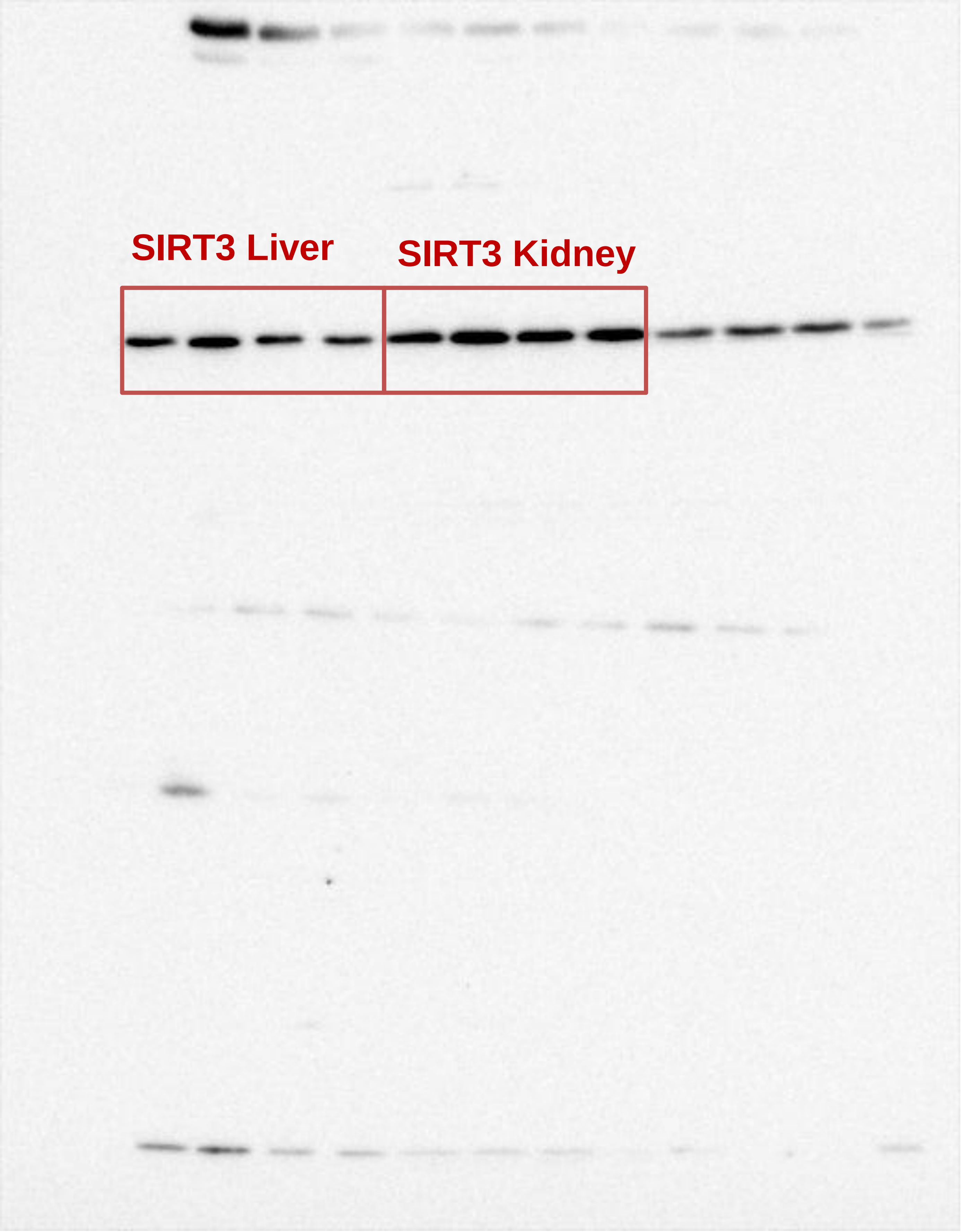

Figure 5A. SIRT6

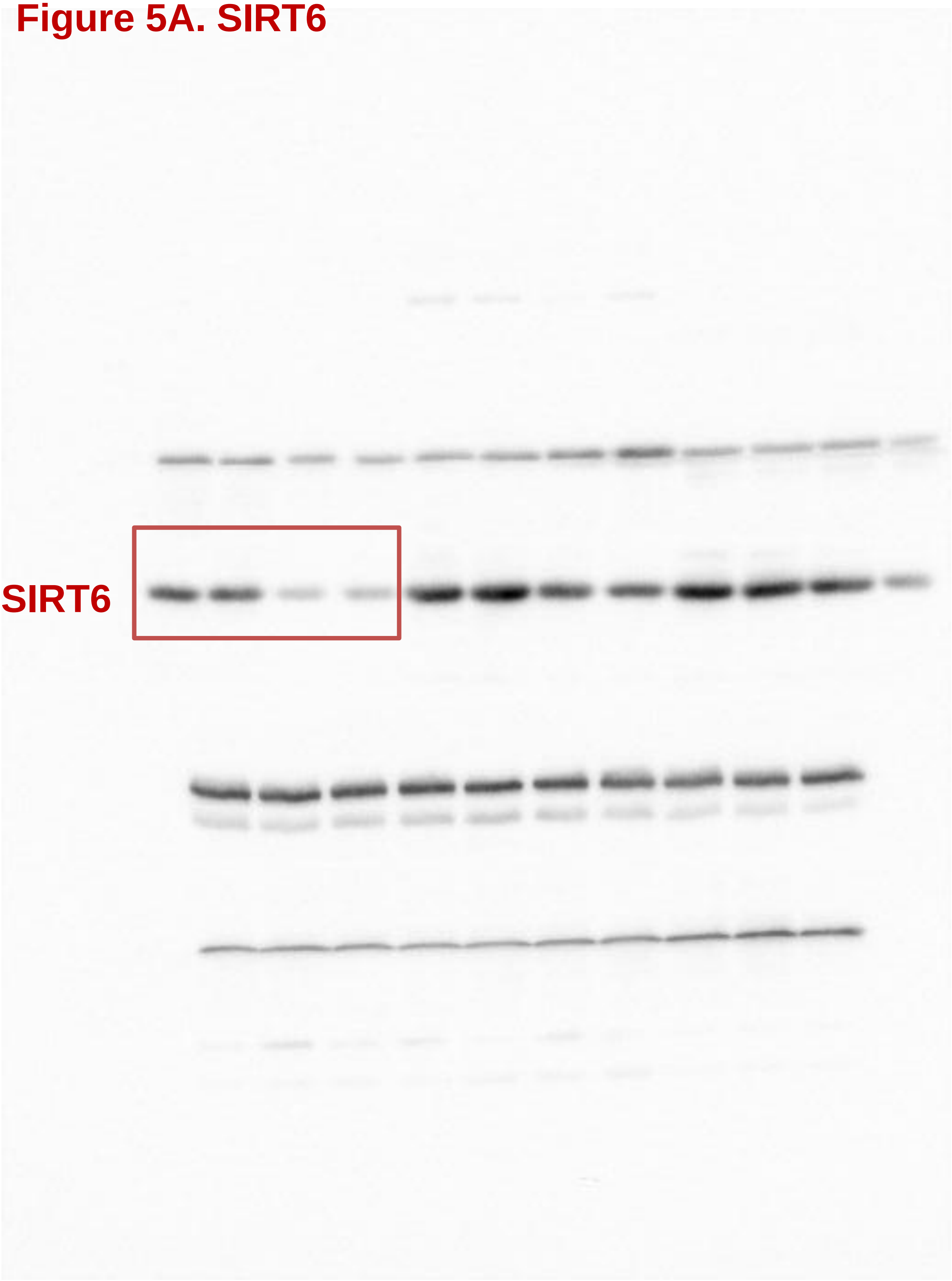

## Figure 5A. SIRT6

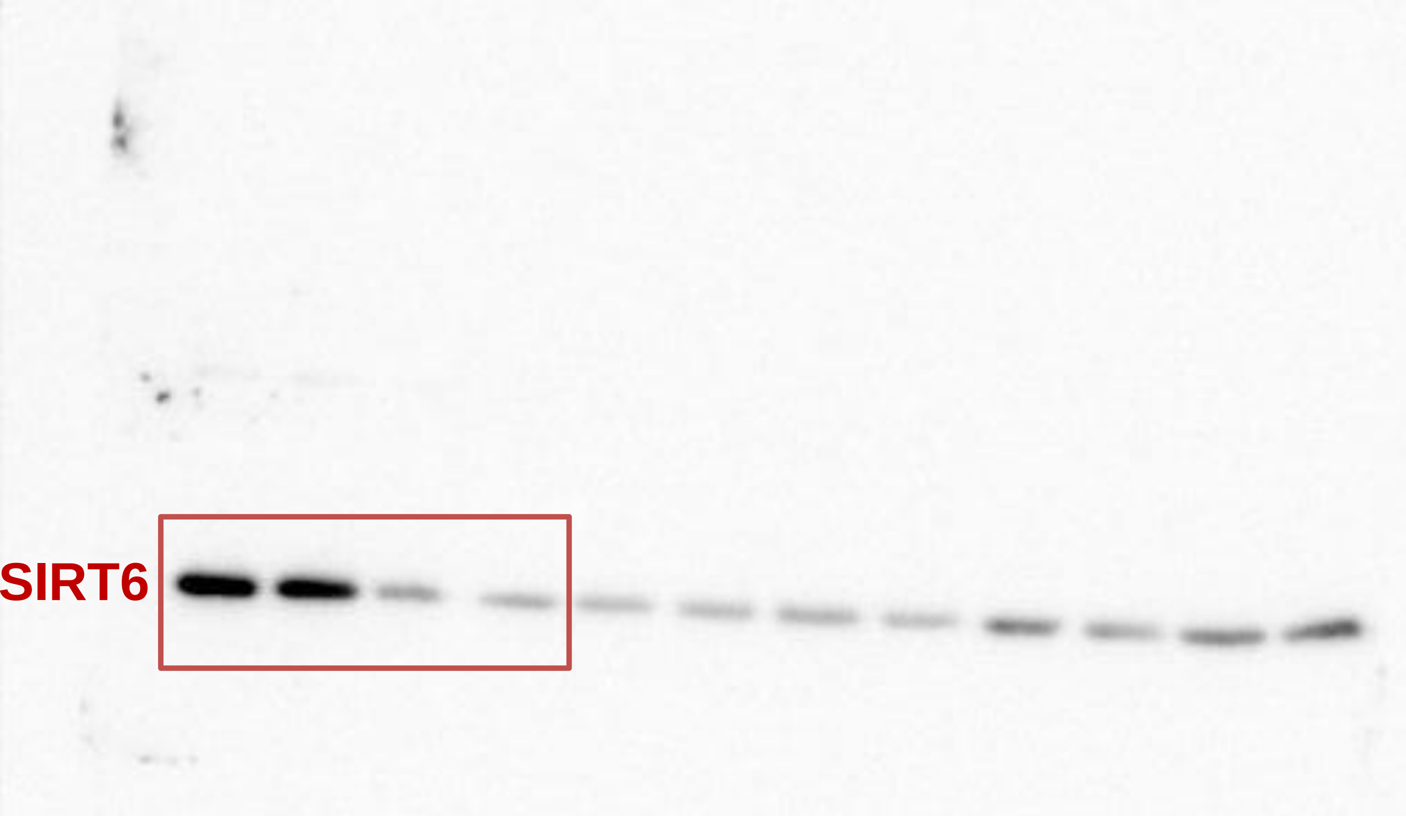

Figure 5A. NF-kB

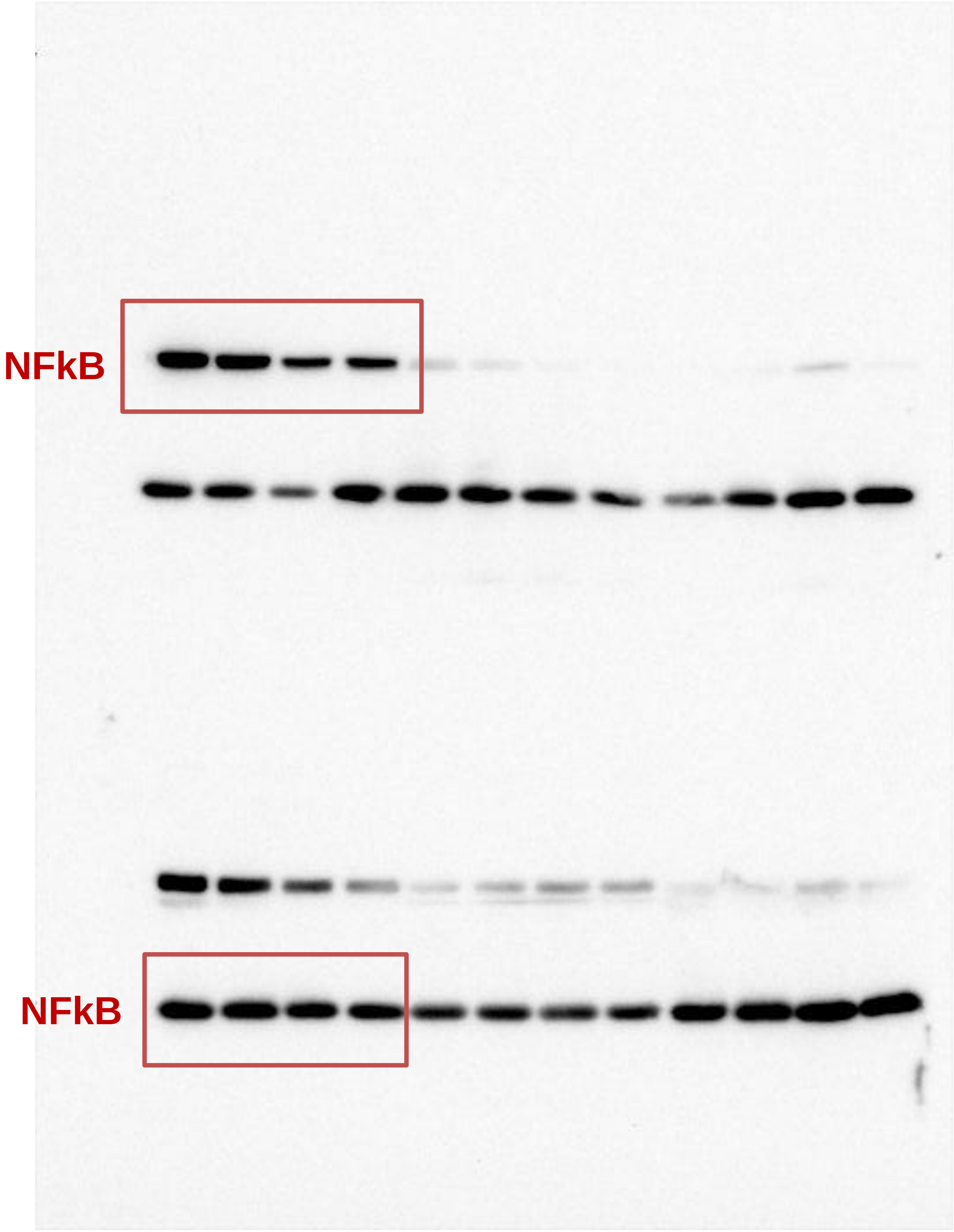

# Figure 5A. Ac-NFkB, Actin

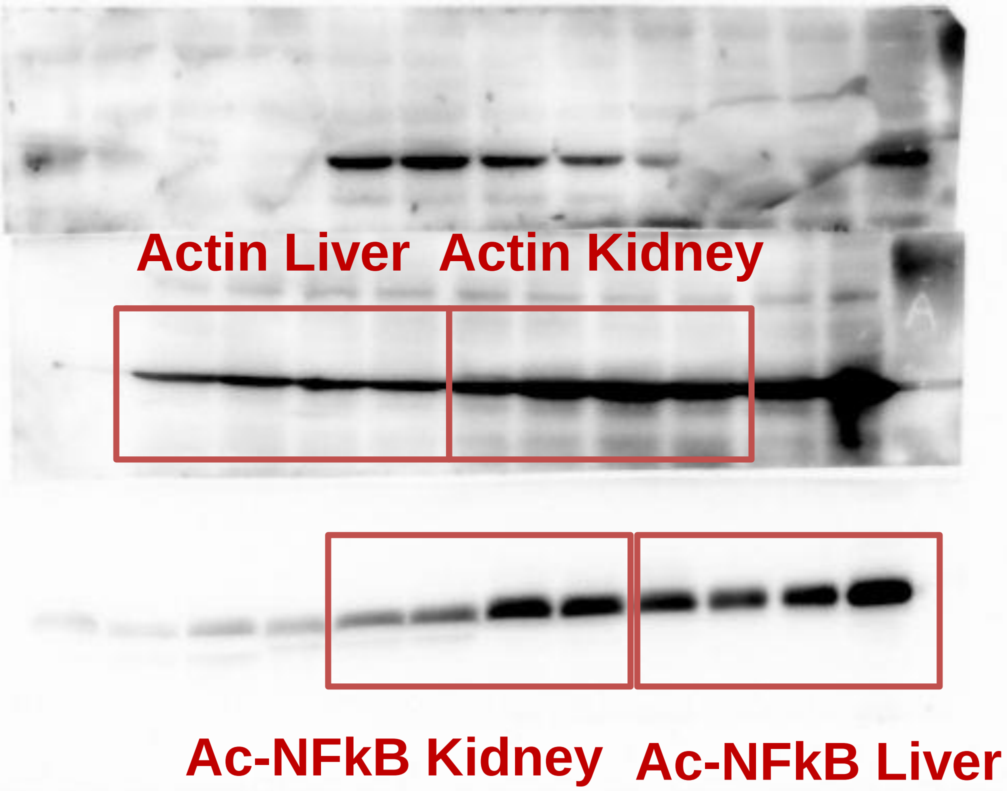

Figure 5B. SIRT6

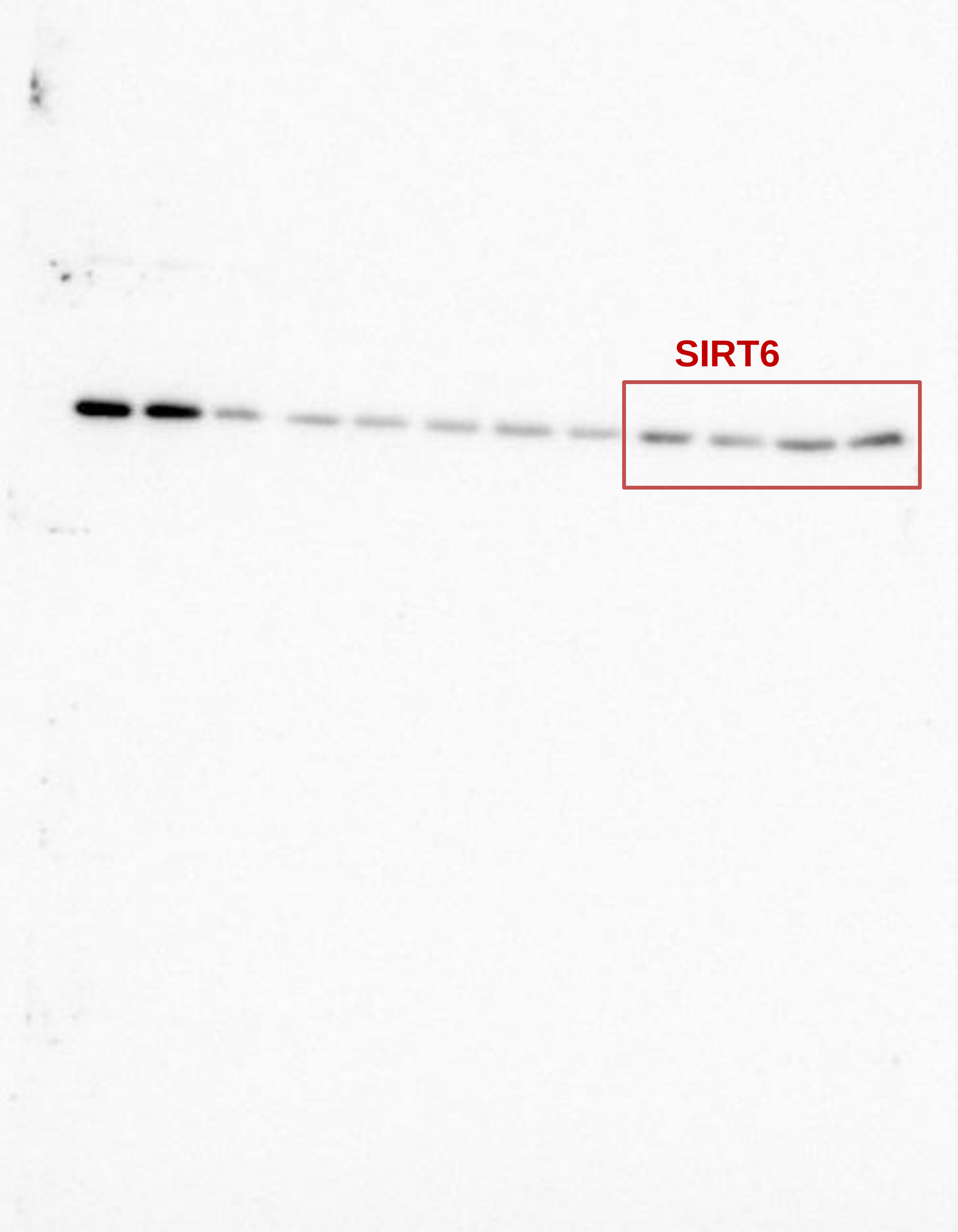

## Figure 5B. SIRT6

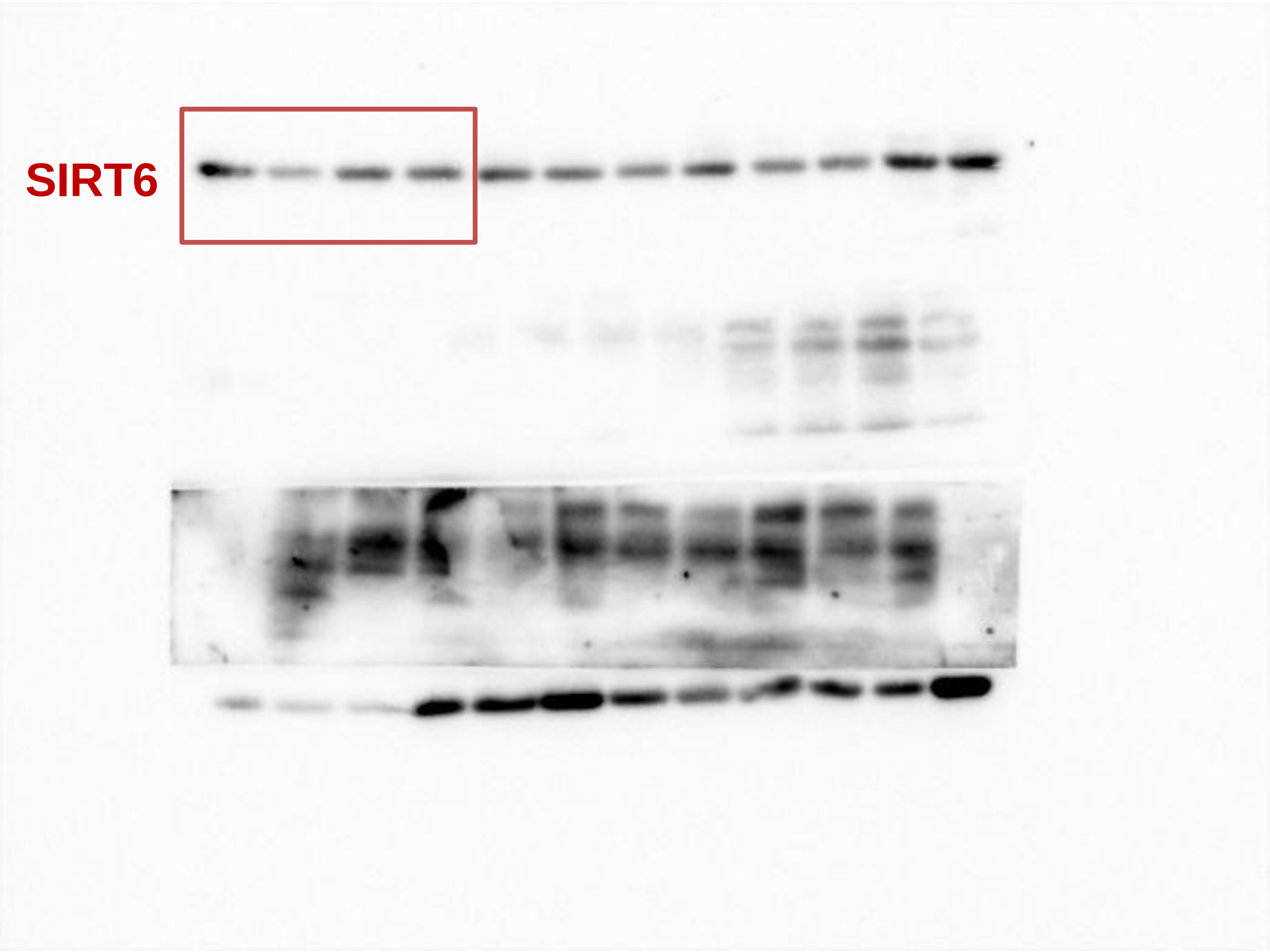

# Figure 5B. NF-kB

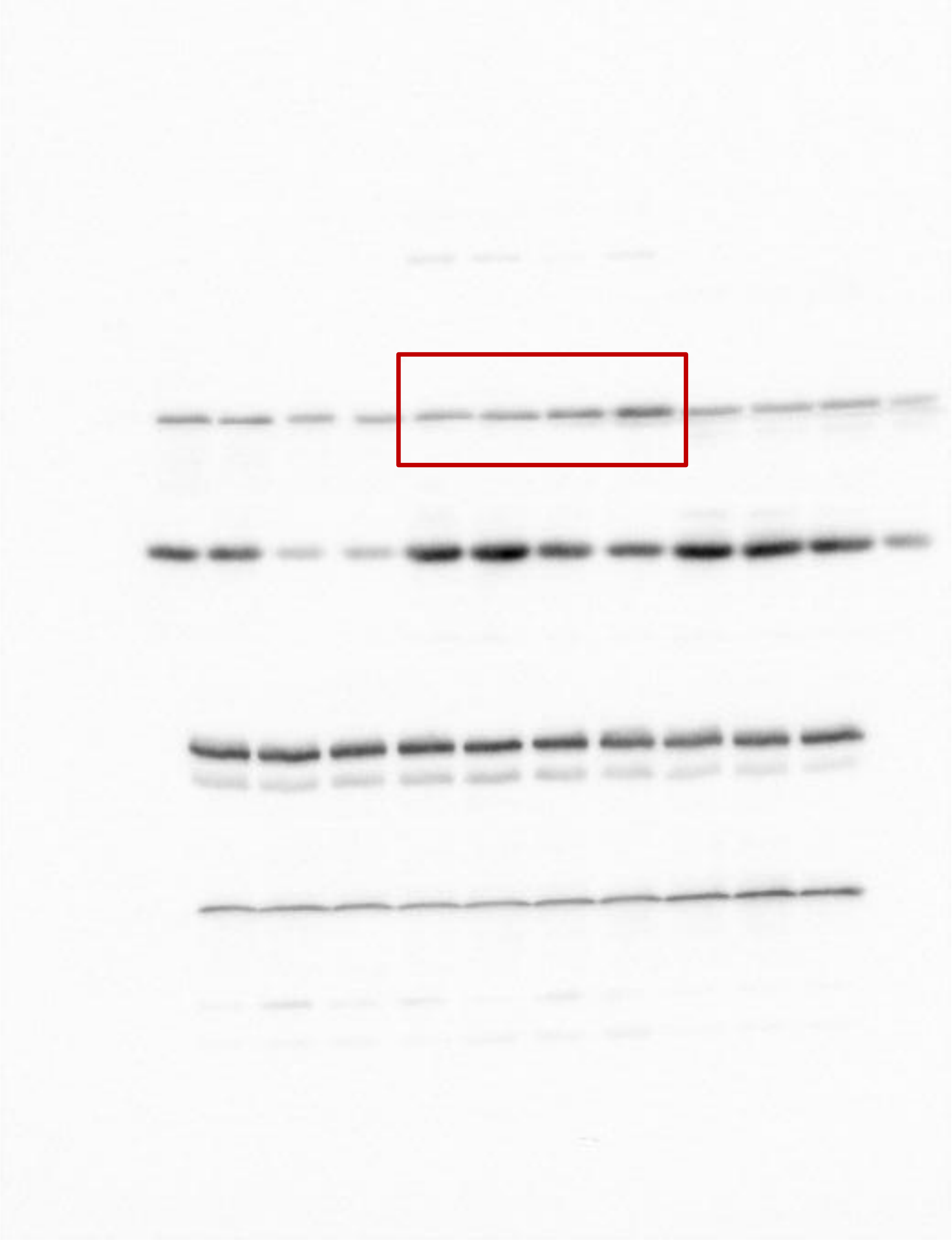

## Figure 5B. Actin

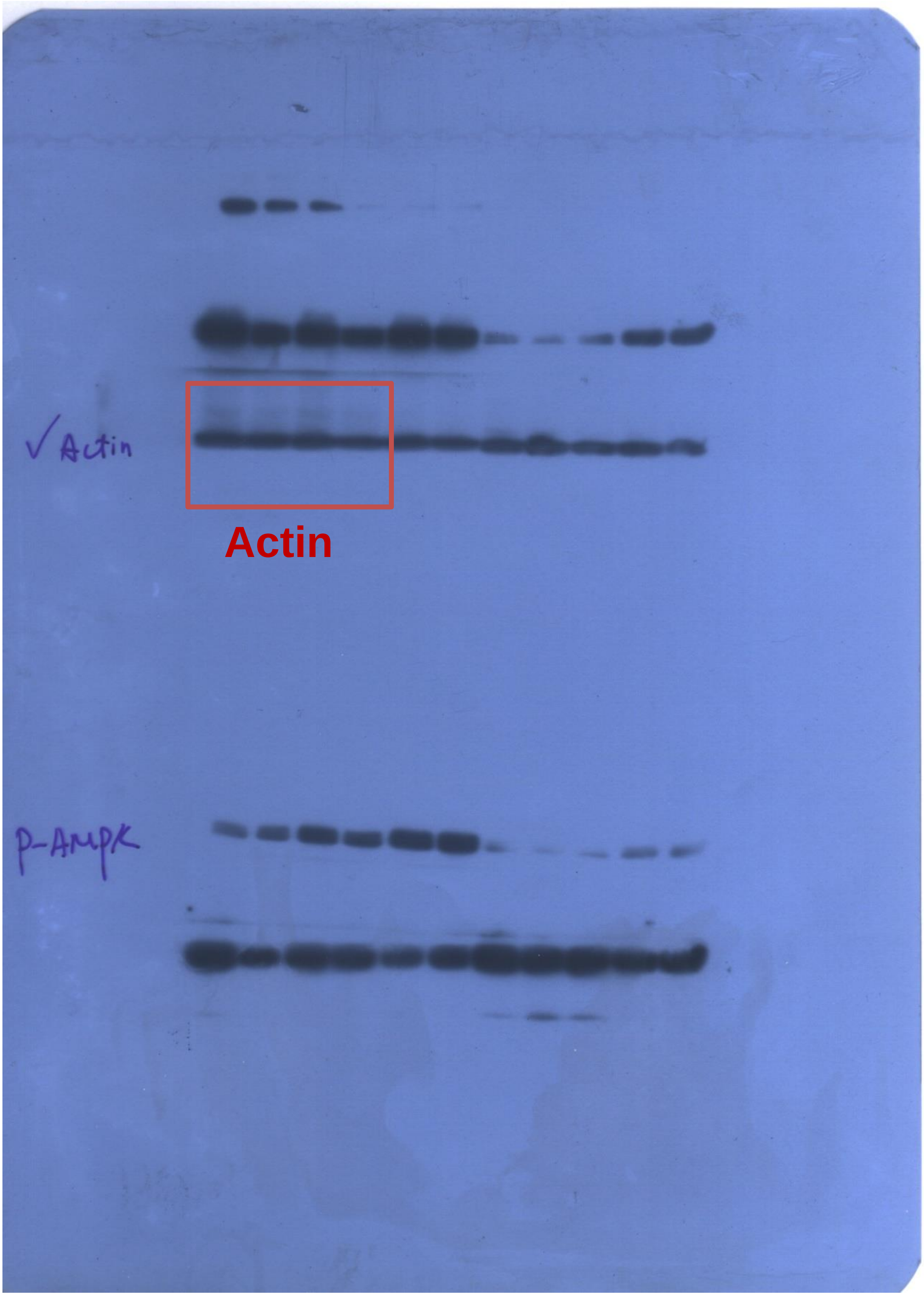

# Figure 6A. Actin

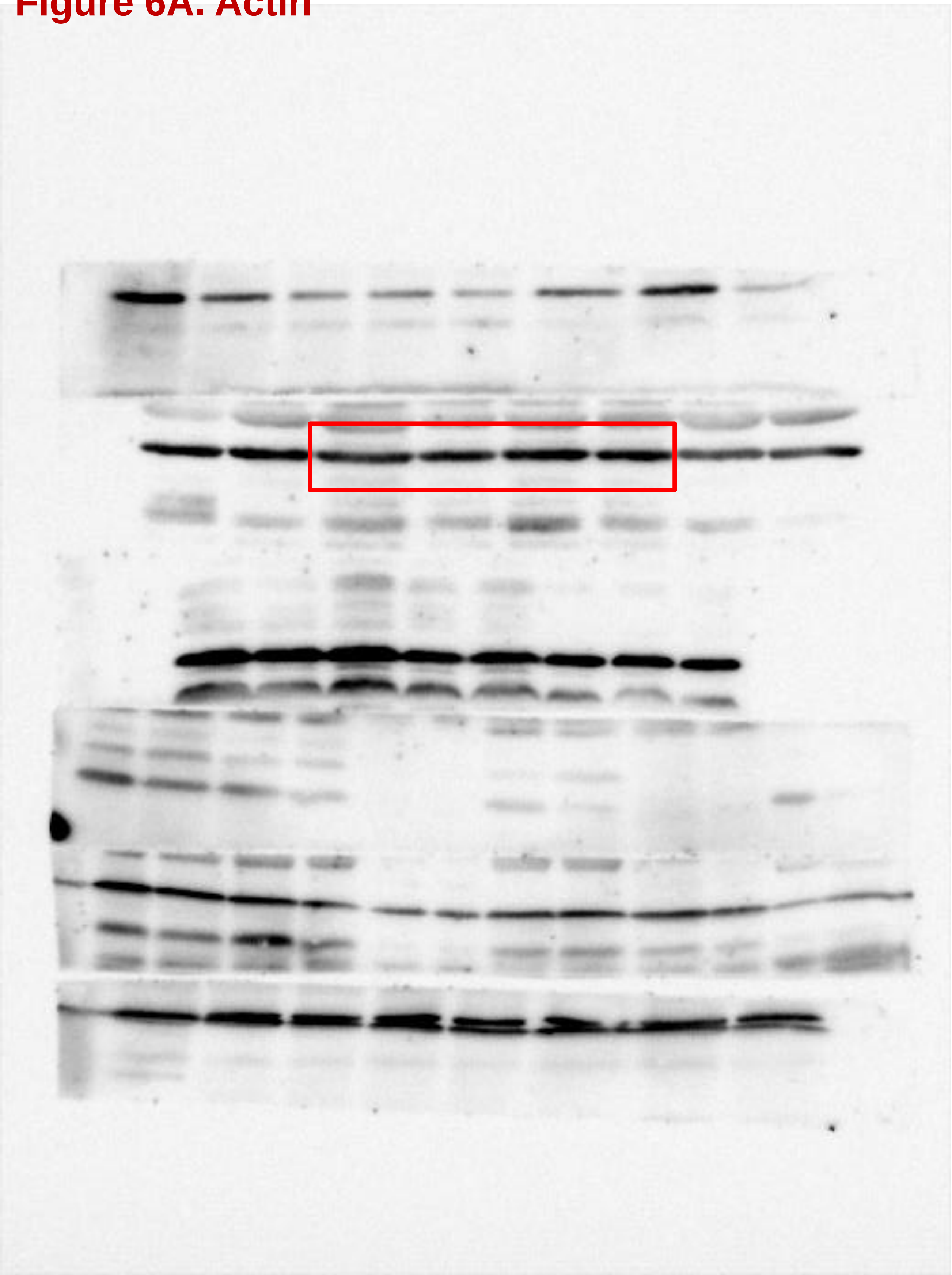

# Figure 6B. Ac-NFkB

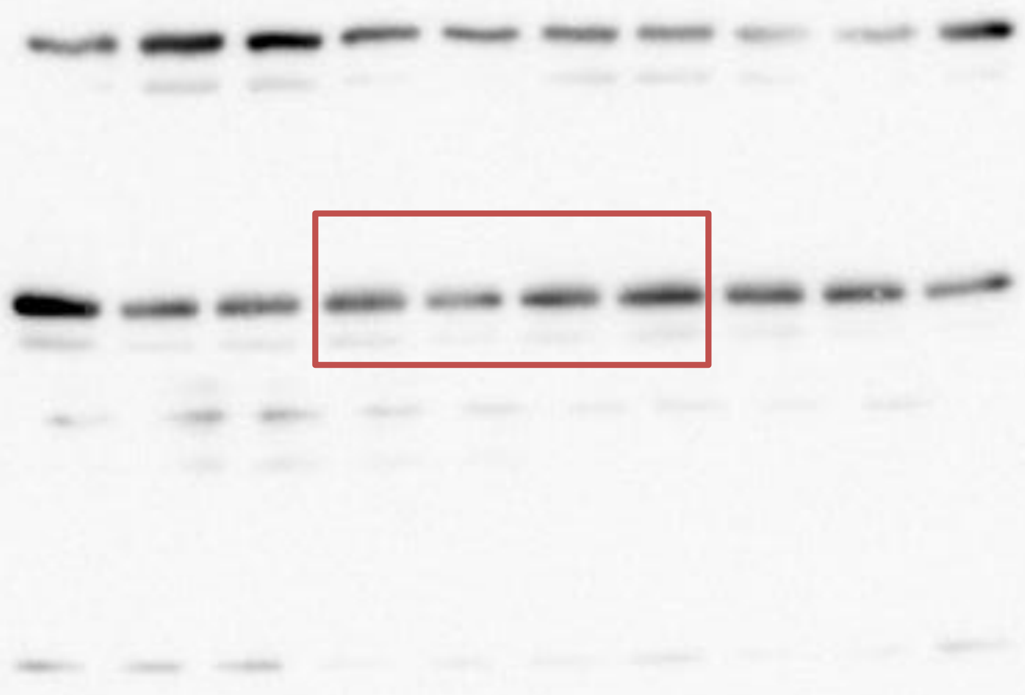

# Figure 6B. NF-kB

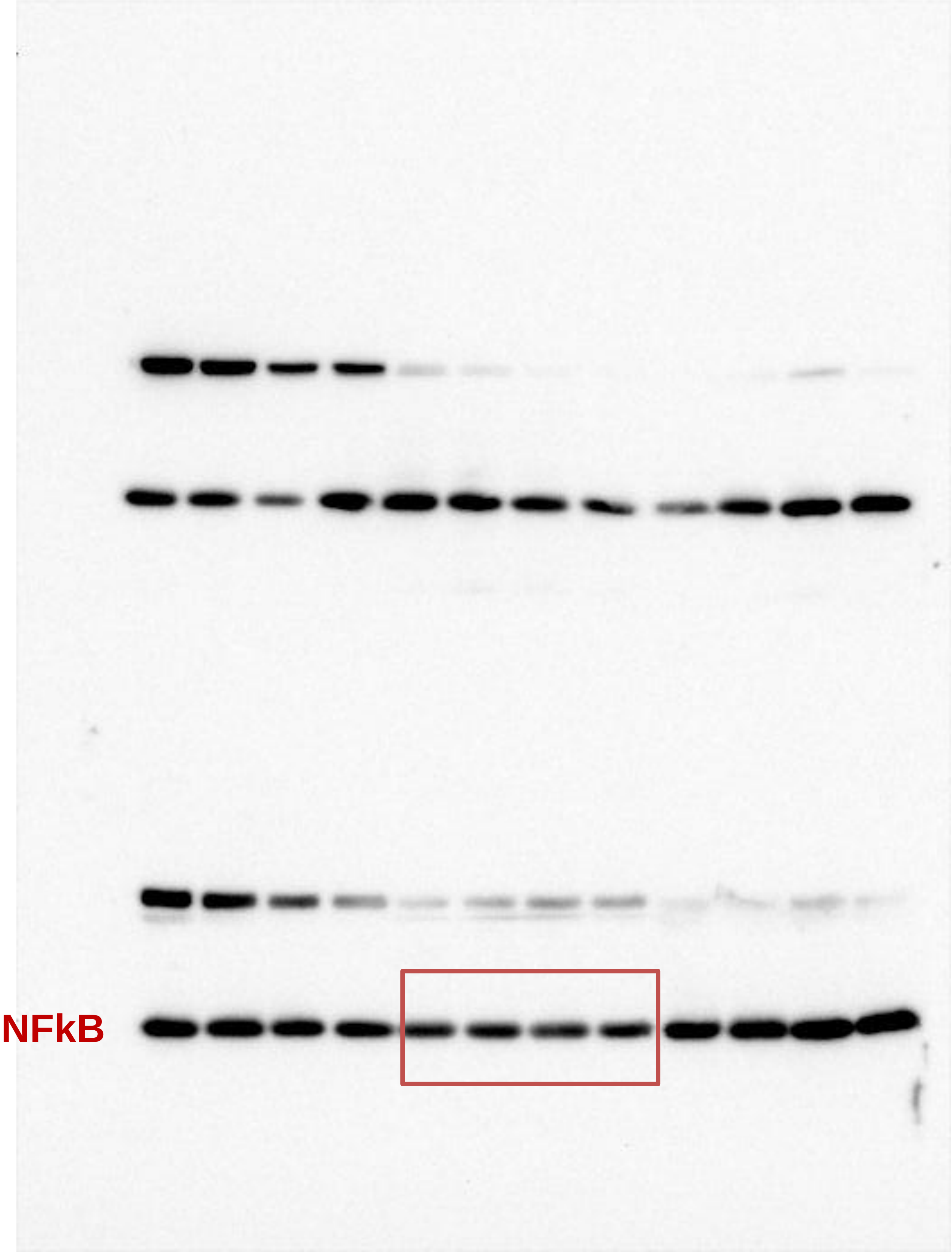

# Figure 6B. NF-kB

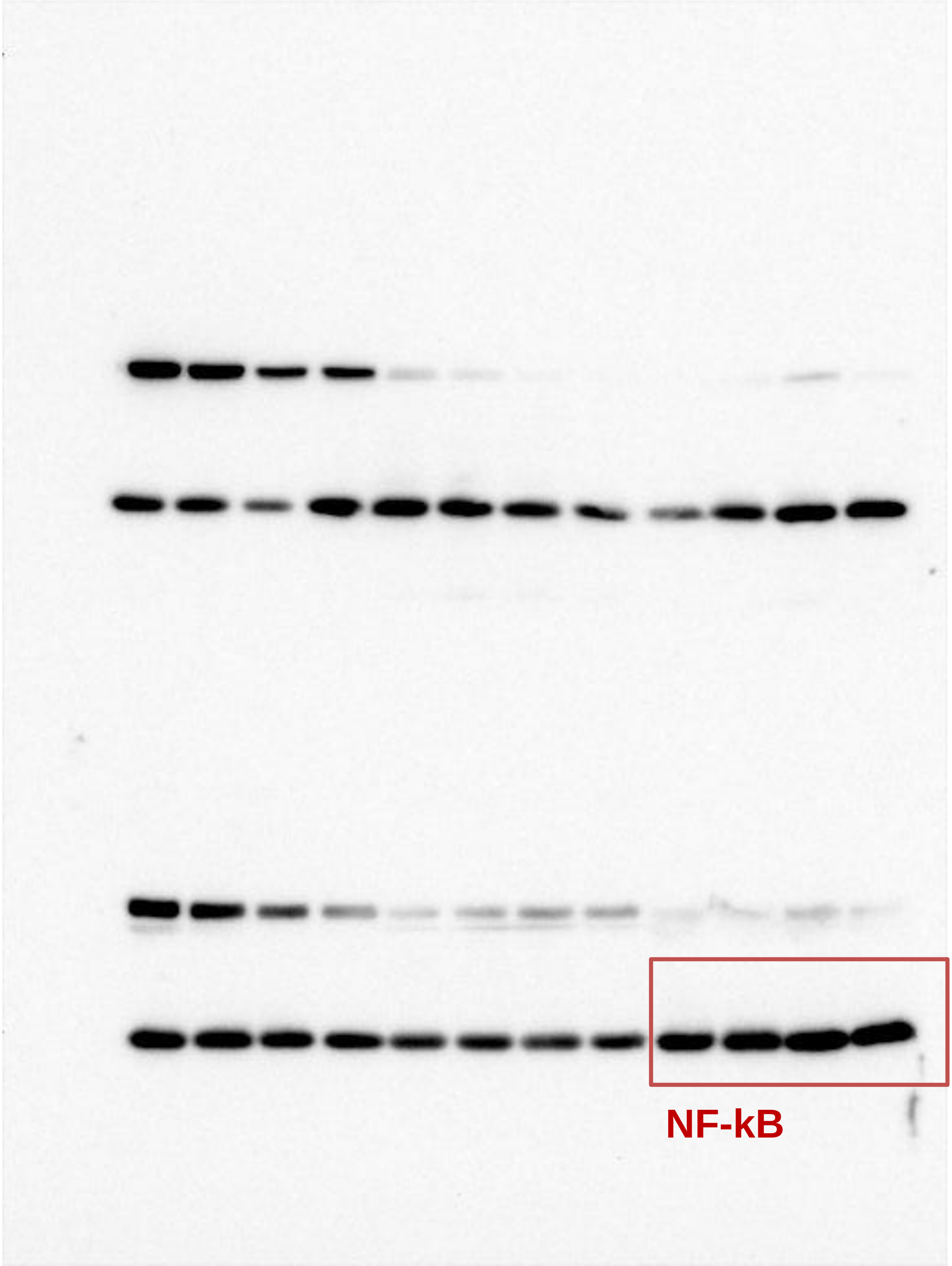

**Figure 6B. SIRT6**

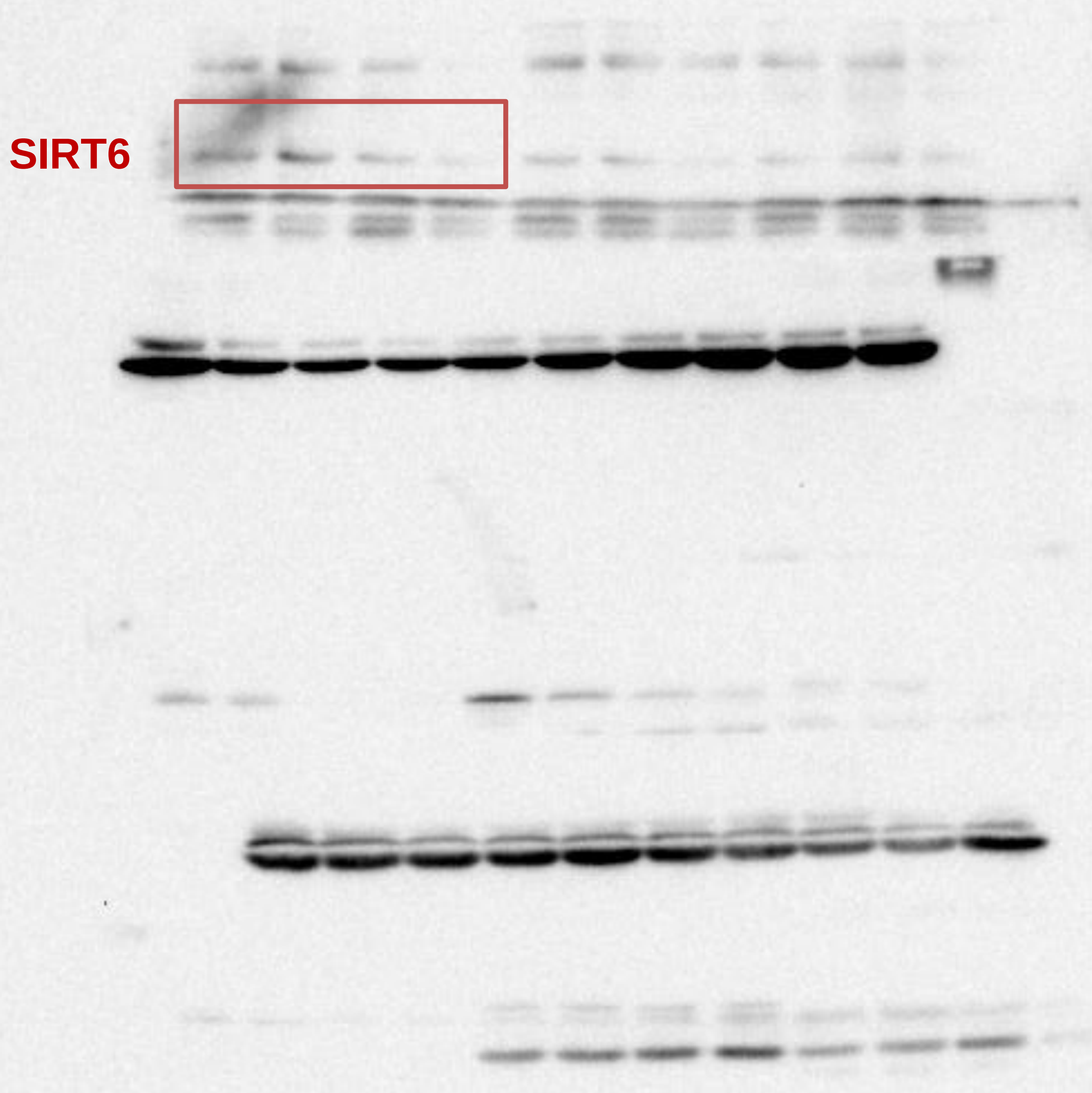

Supplement: Supplementary Figures 3-6 [file aging-10-101559-s003.pdf]
